# Cooperative synaptic and intrinsic plasticity in a disynaptic limbic circuit drive stress-induced anhedonia and passive coping in mice

**DOI:** 10.1038/s41380-020-0686-8

**Published:** 2020-03-11

**Authors:** Marco Pignatelli, Hugo A. Tejeda, David J. Barker, Leonardo Bontempi, Jocelyn Wu, Alejandra Lopez, Sissi Palma Ribeiro, Federica Lucantonio, Eric M. Parise, Angélica Torres-Berrio, Yocasta Alvarez-Bagnarol, Rosa A. M. Marino, Zhao-Lin Cai, Mingshan Xue, Marisela Morales, Carol A. Tamminga, Eric J. Nestler, Antonello Bonci

**Affiliations:** 1 Synaptic Plasticity Section, National Institute on Drug Abuse Intramural Research Program, Baltimore, MD, USA; 2 Neuronal Networks Section, National Institute on Drug Abuse Intramural Research Program, Baltimore, MD, USA; 3 Solomon Snyder Department of Neuroscience, Johns Hopkins School of Medicine, Baltimore, MD, USA; 4 Nash Family Department of Neuroscience, Friedman Brain Institute, Icahn School of Medicine at Mount Sinai, New York 10029 NY, USA; 5 Department of Neuroscience, Baylor College of Medicine, Houston, TX 77030, USA; 6 The Cain Foundation Laboratories, Jan and Dan Duncan Neurological Research Institute at Texas Children’s Hospital, Houston, TX 77030, USA; 7 Department of Psychiatry, University of Texas Southwestern Medical Center, Dallas, TX 75390, USA; 8 Global Institutes on Addictions, Miami, FL, USA; 9 Present address: Department of Psychiatry and Taylor Family Institute for Innovative Psychiatric Research, Washington University School of Medicine, St Louis, MO 63110, USA; 10 Present address: Neuromodulation and Synaptic Integration Unit, National Institute of Mental Health Intramural Research Program, Bethesda, MD, USA

## Abstract

Stress promotes negative affective states, which include anhedonia and passive coping. While these features are in part mediated by neuroadaptations in brain reward circuitry, a comprehensive framework of how stress-induced negative affect may be encoded within key nodes of this circuit is lacking. Here, we show in a mouse model for stress-induced anhedonia and passive coping that these phenomena are associated with increased synaptic strength of ventral hippocampus (VH) excitatory synapses onto D1 medium spiny neurons (D1-MSNs) in the nucleus accumbens medial shell (NAcmSh), and with lateral hypothalamus (LH)-projecting D1-MSN hyperexcitability mediated by decreased inwardly rectifying potassium channel (IRK) function. Stress-induced negative affective states are prevented by depotentiation of VH to NAcmSh synapses, restoring *Kir2.1* function in D1R-MSNs, or disrupting co-participation of these synaptic and intrinsic adaptations in D1-MSNs. In conclusion, our data provide strong evidence for a disynaptic pathway controlling maladaptive emotional behavior.

## Introduction

Stress promotes negative affective states in virtually all neuropsychiatric disorders, including depression, bipolar disorder, and drug addiction [1–5]. Increasing evidence suggests that stress elicits negative affect by perturbing synaptic transmission and intrinsic excitability in various brain regions that subserve emotional and motivational behavior [4, 6, 7]. However, how stress-induced pathological changes in the synaptic and intrinsic processes of neurons affect neuronal signal integration in key nodes of the motivational circuitries involved in adaptive behaviors remains unknown.

The nucleus accumbens (NAc), a key structure of the mesolimbic reward circuit involved in mediating motivational and emotional processes, receives convergent emotional and contextual inputs from limbic structures, such as the ventral hippocampus (VH), and transmits this information to downstream structures, such as the lateral hypothalamus (LH), ventral pallidum (VP), and ventral tegmental area (VTA) [8–10]. The NAc primarily contains GABAergic medium spiny neurons (MSNs) that are dichotomous in their predominant expression of either D1 or D2 dopamine receptors (D1-MSNs and D2-MSNs, respectively) [11, 12], and whose role in regulating circuit function may differ based on the discrete subregions in which they are embedded [13, 14]. The NAcc can be further subdivided into the NAc core and shell, with the latter being considered part of the extended amygdala [15].

Although evidence has shown that stress induces both synaptic and intrinsic adaptations within the NAc [16–18] and that D1 and D2 subpopulations of the NAc have specific functional effects on stress-mediated behaviors [19–22], many questions remain open. Do stress-induced alterations in both NAc synaptic and intrinsic mechanisms contribute to anhedonia and passive coping? Is a specific cell type within the NAc responsible for driving stress-induced negative affect? Does stress-induced plasticity in synaptic transmission and intrinsic excitability participate in modifying the information flow into and out of the NAc? To address these questions, we performed ex vivo cell-type-specific electrophysiological recordings within the NAc medial shell (NAcmSh) from mice that developed stress-induced negative affect, together with in vivo fiber photometry, neural circuit tracing, and viral/transgenic manipulation experiments. Here, we show that stress drives a negative affective state, as operationally defined by increased anhedonia and passive coping, and that this maladaptive behavior is mediated by co-participation of both synaptic transmission at VH to NAcmSh D1-MSN synapses and intrinsic hyperexcitability of D1-MSNs that project to the LH. Our findings describe a previously unappreciated role of synaptic and intrinsic plasticity in NAc D1-MSNs in mediating negative affective states.

## Results

### Repeated stress exposure induces negative affective states in mice

**Fig. 1:**
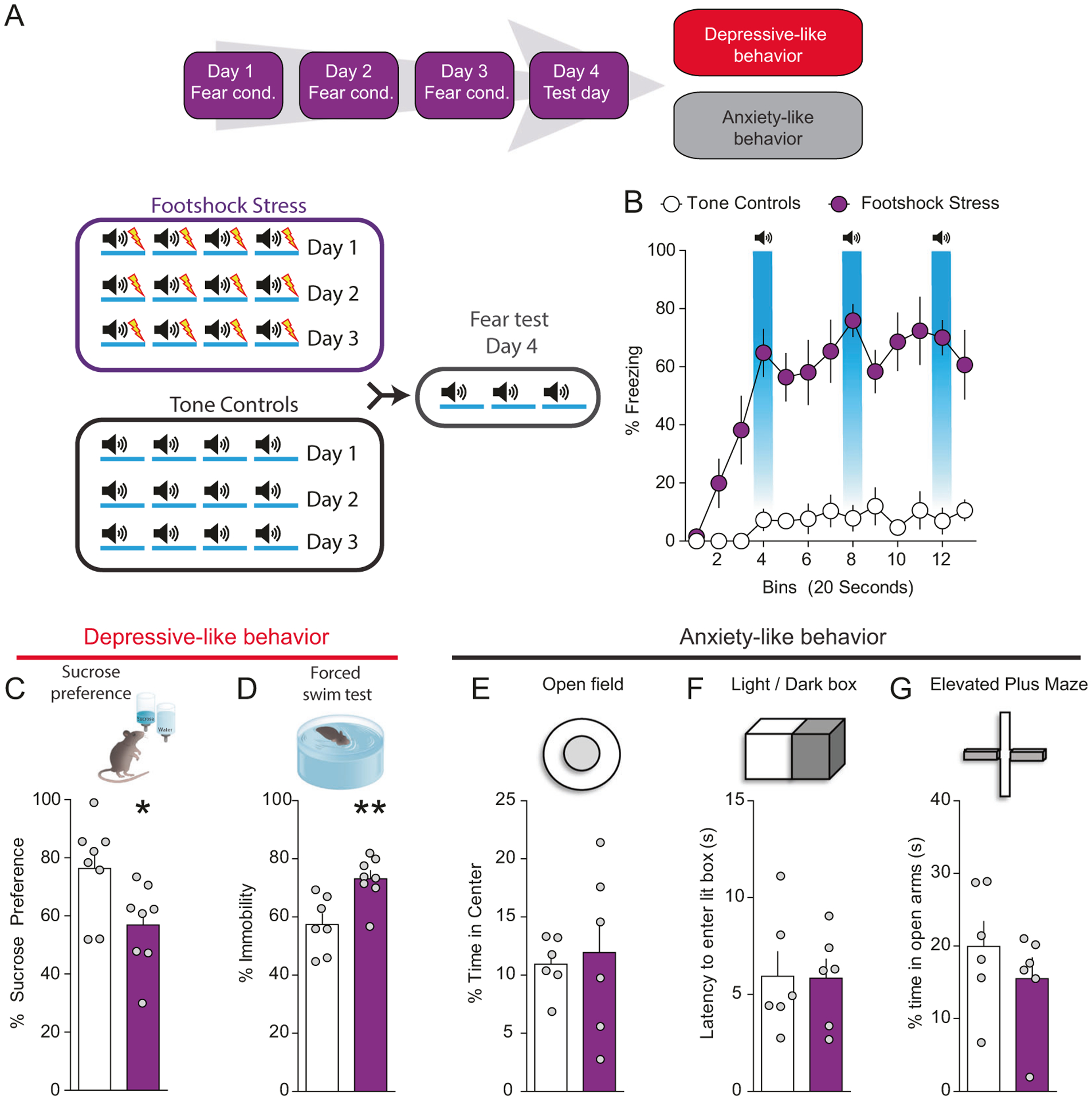
Repeated footshock stress drives anhedonia and passive coping, but not anxiety-like behavior. **a** Timeline of fear conditioning, sucrose preference, and forced swim test. Schematic of fear conditioning procedures. **b** Conditioned stimuli predictive of fear promote freezing behavior (*n* = 8 per group; two-way ANOVA; treatment × time interaction; ANOVA; *F*(_24,252_) = 6.29, *p* < 0.0001). **c** Footshock stress decreases sucrose preference (*n* = 8 per group; *t*-test; *t*_(14)_ = 2.52, *p* = 0.025). **d** Footshock stress increases immobility in the forced swim test (*n* = 7–8 per group; *t*-test; *t*_(13)_ = 3.49, *p* = 0.004). **e** No significant difference in the amount of time spent in the center of the open field maze between footshock and tone groups (*n* = 6 per group; *t*-test; *t*_(10)_ = 0.32, *p* = 0.75). **f** Latency to enter dark chamber in the light-dark box test does not differ between footshock and tone groups (*n* = 6 per group; *t*-test; *t*_(10)_ = 0.06, *p* = 0.95). **g** Time spent in the open arms of the elevated plus maze does not differ between footshock and tone groups (*n* = 6 per group; *t*-test; *t*_(10)_ = 0.99, *p* = 0.35).

In our first series of experiments, we conditioned mice to associate an auditory stimulus with a mild footshock over 3 days (footshock stress group; Fig. 1a). Control mice received the auditory stimulus without the footshocks (tone group; Fig. 1a). On day 4, freezing (an index of fear-related behavior) to the context and to the footshock-predictive cue was assessed (Fig. 1b). Mice in the footshock stress group showed high levels of freezing compared with mice in the tone group that did not display any level of freezing (Fig. 1b). We subsequently determined whether footshock stress-induced maladaptive passive coping mechanisms and deficits in reward function (i.e., anhedonia). Mice that underwent footshock stress exhibited decreased sucrose preference (a behavioral measure of anhedonia-like behavior) (Fig. 1c) and increased immobility in the forced swim test (FST, a passive response to stress that is interpreted as a sign of passive coping) relative to control mice (Fig. 1d), suggesting that repeated aversive experiences drive both anhedonia and passive coping. To determine whether footshock stress also induced generalized anxiety-like behaviors, a separate cohort of mice was tested in a battery of tests widely used for measuring this behavioral domain following tone or footshock stress exposure. Repeated stress failed to modify anxiety-like behaviors in the open field (Figs. 1e and S1), light/dark box box (Figs. 1f and S1) and elevated plus maze (Figs. 1g and S1). These results suggest that repeated footshock stress specifically promotes negative affective states lacking generalized anxiety-related features in mice. Anxiety develops in anticipation to an imminent threat or the perception of a threat, even if the threat is impossible or unlikely. Since our stress paradigm consisted solely of predictable shocks, it is possible that lack of uncertainty produced negative affect without modifying anxiety-like behavior. As suggested by preclinical and clinical evidence, anxiety is triggered by unpredictability and uncertainty about a possible future threat, which disrupt the ability to mitigate its negative emotional impact [23, 24].

### VH to NAc synapses are actively recruited in vivo during aversive learning

**Fig. 2:**
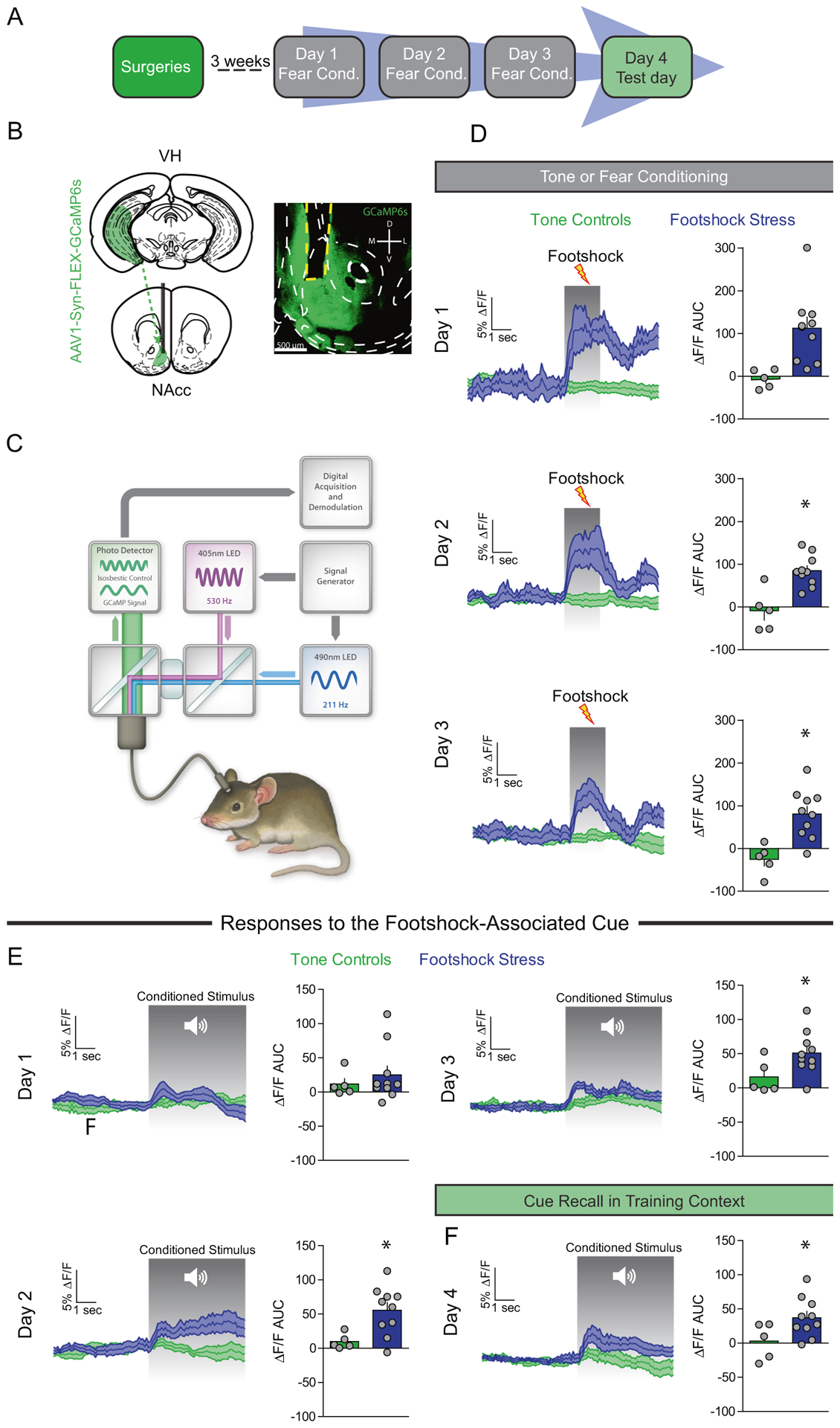
VH afferents are recruited by footshock stress and stress-predictive cues. **a** Experimental timeline. In vivo fiber photometry recordings were conducting during conditioning and fear recall procedures (days 1–4). **b** Schematic depicting AAV1-Syn-GCaMP6s injection into the VH and optical fiber implantation into the NAc of WT mice. Representative image of optical fiber and terminal GCaMP6 expression in the NAc from a mouse injected with AAV1-Syn-GCaMP6s into the VH. **c** Schematic of fiber photometry system. **d** Footshock-induced VH terminal Ca^2+^ activity in NAc over the course of the three days conditioning procedures. Left: time course of VH terminal Ca^2+^ dynamics during a 5 s window before and after a footshock. Right: area under the curve (AUC) of the Δ*F*/*F*. Day 1 (*t*-test; *t*_(13)_ = 3.11, *p* = 0.008). Day 2 (*t*-test; *t*_(13)_ = 4.23, *p* = 0.001). AUC day 3 (*t*-test; *t*_(13)_ = 3.77, *p* = 0.002). **e** Cue-induced Ca^2+^ activity during tone or fear conditioning. Left: mean Ca^2+^ Δ*F*/*F* 5 s before and after the onset of the 20 s auditory cue. Right: AUC of the Δ*F*/*F*. AUC day 1 (*n* = 5–10; *t*-test; *t*_(13)_ = 0.70, *p* = 0.50). AUC day 2 (*t-*test; *t*_(13)_ = 2.78, *p* = 0.02). AUC day 3 (*t*-test; *t*_(13)_ = 2.18, *p* = 0.048). **f** Cue-mediated Ca^2+^ activity during fear recall in the training context. Right: AUC day 4 (*t*-test; *t*_(13)_ = 2.21, *p* = 0.046).

The VH is the largest limbic glutamatergic input to the NAcmSh and has been implicated in mediating motivationally-relevant behavior [8, 25, 26]. Thus, we first determined whether activity dynamics of the VH-to-NAc projection could encode footshock stress and threat-predictive stimuli. To this end, we utilized fiber photometry recordings of VH afferents to the NAcmSh in mice. AAV expressing the genetically-encoded Ca^2+^ indicator, GCaMP6s, was injected into the VH and an optical fiber was implanted above the NAcmSh to monitor VH terminal Ca^2+^ dynamics in vivo (Figs. 2a–c and S2). On day 1 of footshock stress, robust increases in VH terminal Ca^2+^ activity were observed in response to footshocks (Fig. 2d). Interestingly, with repeated cue-footshock associations, cue-evoked VH terminal Ca^2+^ responses evolved (Fig. 2e). On the test day, there were increased VH terminal Ca^2+^ transients when the auditory cue alone without the footshock was presented to the mice (Fig. 2f). These results suggest that VH afferents to the NAcmSh innately encode footshocks as well as footshock-predictive cues after learning. These data provide a basis wherein repeated footshock stress activates VH afferents to the NAcmSh. However, it is not known whether engagement of VH afferents during footshock stress results in plasticity of VH synaptic transmission to D1- and D2-MSNs.

### VH afferents are selectively potentiated onto NAcmSh D1-MSNs after stress

**Fig. 3:**
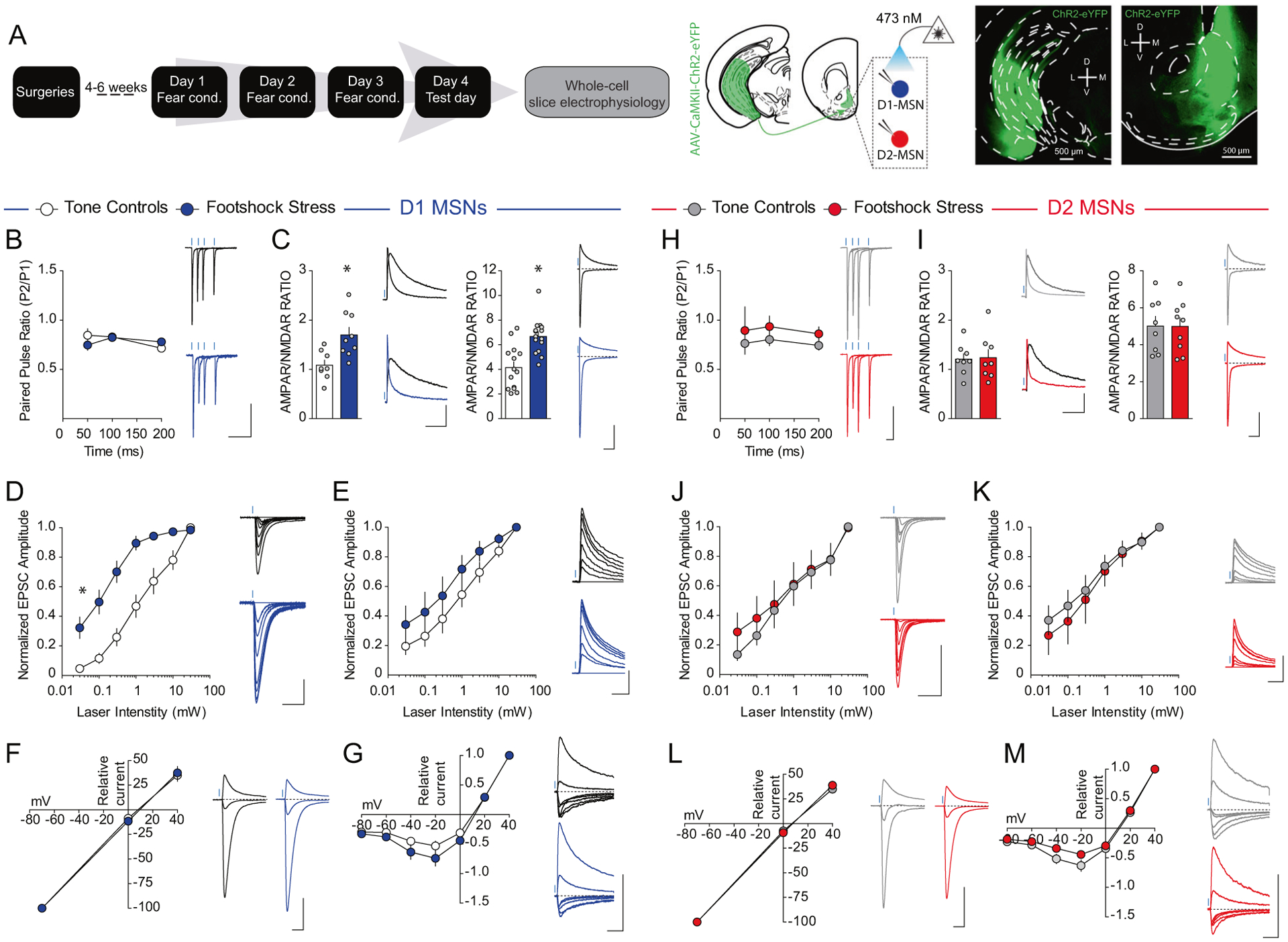
VH synapses onto D1-, but not D2-, MSNs are potentiated with footshock stress. **a** Experimental timeline. Representative image of ChR2-eYFP expression in the VH (left) and NAc (right) of D1-tdtomato mice. **b**, **h** Paired pulse ratio is unaffected by conditioned fear in D1-MSNs (*n* = 8 per group; two-way ANOVA; treatment main effect; *F*_(2, 18_) = 0.06, *p* = 0.9388; ISI main effect; *F*_(2,_ 36) = 3.44, *p* < 0.043; treatment × intensity interaction; *F*_(4, 36_) = 2.21, *p* = 0.087) and D2-MSNs (*n* = 6 controls; *n* = 9 footshock stress; two-way ANOVA; treatment main effect; *F*_(2, 18_) = 0.37, *p* = *0.6983*; ISI main effect; *F*_(2, 36_) = 0.53, *p* = *0.5917*; treatment × intensity interaction; *F*_(4, 36_) = 0.07, *p* = 0.9912). **c**, **i** Biophysically-isolated AMPA-R/NMDAR in D1-MSNs (left; blue) and D2-MSNs (right; red) in mice in the tone (white), and footshock (light color) groups. D1-MSNs (*n* = 14–15 cells per group; *t*-test; *t*_(27)_ = 4.26, *p* = 0.0002). D2-MSNs (*n* = 8–9 per group; *t*-test; *t*_(15)_ = 0.03, *p* = 0.98). Pharmacologically isolated AMPA-R/NMDAR in D1-MSNs and D2-MSNs. D1-MSNs (*n* = 8–9 per group; *t*-test; *t*_(15)_ = 3.24, *p* = 0.0055). D2-MSNs (*n* = 8 per group; *t*-test; *t*_(14)_ = 0.15, *p* = 0.88). **d**, **j** Increased VH AMPAR oEPSCs in D1-MSNs from mice in the footshock group relative to the tone group (*n* = 7–9 per group; two-way ANOVA; treatment × intensity; *F*_(6, 84)_ = 8.2, *p* < 0.0001). VH AMPAR oEPSC input curves in D2-MSNs are not significantly different between tone and footshock groups (*n* = 6–8 per group; two-way ANOVA; treatment main effect; *F*_(1, 12_) = 0.12, *p* = 0.73; Intensity main effect; *F*_(6, 72)_ = 34.26, *p* < 0.0001; Treatment × intensity interaction; *F*(_6,72_) = 0.44, *p* = 0.85). **e**, **k** No difference in VH NMDAR oEPSCs in D1-MSNs (*n* = 7–8 per group; two-way ANOVA; treatment main effect; *F*_(1, 13)_ = 1.48, *p* = 0.2449; intensity main effect; *F*_(6, 78)_ = 63.61, *p* < 0.0001; treatment × intensity interaction; *F*(_6,78_) = 0.79, *p* = 0.5811), and D2-MSNs between footshock and tone groups (*n* = 5–9 per group; two-way ANOVA; treatment main effect; *F*_(2, 19)_ = 0.25, *p* = 0.7798; intensity main effect; *F*_(6, 114)_ = 68.1, *p* < 0.0001; treatment × intensity interaction; *F*_(12, 114)_ = 0.24, *p* = 0.9957). **f**, **l** Current-voltage curves of VH AMPAR oEPSCs. D1-MSNs (*n* = 8–11 per group; Two-way ANOVA; treatment main effect; *F*_(1, 17)_ = 0.002, *p* = 0.96; voltage main effect; *F*_(2, 34)_ = 566.99, *p* < 0.0001; treatment × voltage interaction; *F*_(2, 34)_ = 0.2, *p* = 0.82). D2-MSNs (*n* = 8–10 per group; two-way ANOVA; treatment main effect; *F*_(1, 16)_ = 0.15, *p* = 0.70; voltage main effect; *F*_(2, 32)_ = 1241.32, *p* < 0.0001; treatment × voltage interaction; *F*_(2, 32)_ = 0.56, *p* = 0.58). **g**, **m** Current-voltage curves of VH NMDAR oEPSCs. D1-MSNs (*n* = 10 per group; two-way ANOVA; treatment main effect; *F*_(1, 18)_ = 2.14, *p* = 0.16; voltage main effect; *F*_(6,108)_ = 244.69, *p* < 0.0001; treatment × voltage interaction; *F*_(6,108)_ = 1.36, *p* = 0.24). D2-MSNs (*n* = 8–10 per group; two-way ANOVA; treatment × voltage interaction; *F*_(6,96)_ = 2.37, *p* = 0.035).

To determine whether recruitment of VH afferents to the NAc by stress induces plastic changes in VH inputs to D1-and D2-MSNs in the NAcmSh, we conducted ex vivo whole-cell electrophysiological recordings from D1-TdTomato and A2A-Cre-TdTomato in the medial portion of the NAc shell in mice injected with AAV_1_-CaMKII-ChR2-eYFP into the VH (Fig. 3a). We first determined whether there were any adaptations in presynaptic probability of release by examining the paired pulse ratio (PPR) of VH-evoked optogenetically-evoked excitatory postsynaptic currents (VH-oEPSCs). PPR of VH-oEPSCs did not differ between stressed and control mice in both D1-and D2-MSNs (Fig. 3b, h). Next, we determined whether footshock stress modified the strength of excitatory VH synapses onto D1- and D2-MSNs postsynaptically by determining the α-amino-3-hydroxy-5-methyl-4-isoxazolepropionic acid receptor (AMPAR) to N-methyl-D-aspartate receptor (NMDAR) ratio. Biophysical isolation of AMPAR and NMDAR currents revealed an enhanced AMPAR/NMDAR in D1-MSNs from stressed mice relative to tone controls (Fig. 3c). Enhanced synaptic strength of VH inputs to D1-MSNs was also evident in stressed mice when the AMPAR/NMDAR was determined by pharmacologically isolating AMPAR and NMDAR currents using the NMDAR antagonist, AP-V. No change in VH synaptic strength was observed in D2-MSNs (Fig. 3i). Increased AMPAR/NMDAR suggests an enhanced synaptic strength in D1-MSNs after stress. However, it is not clear whether increased AMPAR/NMDAR is due to increased AMPAR currents, decreased NMDAR currents, or a combination of both. To answer these questions, we determined whether footshock stress modified AMPAR and/or NMDAR currents by examining input–output relationships of pharmacologically isolated AMPAR and NMDAR currents in D1-and D2-MSNs. D1-MSNs in stressed mice showed enhanced AMPAR currents relative to tone controls (Fig. 3d). This effect was absent in D2-MSNs (Fig. 3j). Moreover, NMDAR current input–output curves did not significantly differ between stress and control groups in both D1- and D2-MSNs (Fig. 3e, k). These results suggest that footshock stress selectively strengthens VH synapses to D1-MSNs by increasing AMPAR function and/or number. Repeated footshock stress did not modify ChR2-eYFP afferent innervation of the NAcmSh suggesting that increased AMPAR currents after footshock stress is not due to increased VH afferents (Fig. S3). We next determined whether stress-induced changes in AMPAR currents were due to changes in AMPAR subunit composition by determining the current–voltage (*I*/*V*) relationship of AMPAR currents. AMPAR *I/V* curves did not differ between groups in both D1- and D2-MSNs (Fig. 3f, l), suggesting that footshock-induced enhancement of AMPAR currents in D1-MSNs is not due to a change in AMPAR subunit composition. Moreover, NMDAR *I/V* curves did not significantly differ between groups in D1- and D2-MSNs (Fig. 3g, m), suggesting that NMDARs also do not undergo gross change in subunit composition or voltage dependence. Collectively, these results suggest that repeated stress selectively engages VH afferents to the NAc and this results in strengthening of VH synapses onto NAcmSh D1-MSNs by increasing AMPAR function and/or expression.

### Depotentiation of VH afferents to the NAcmSh reverses stress-induced anhedonia and passive coping

**Fig. 4:**
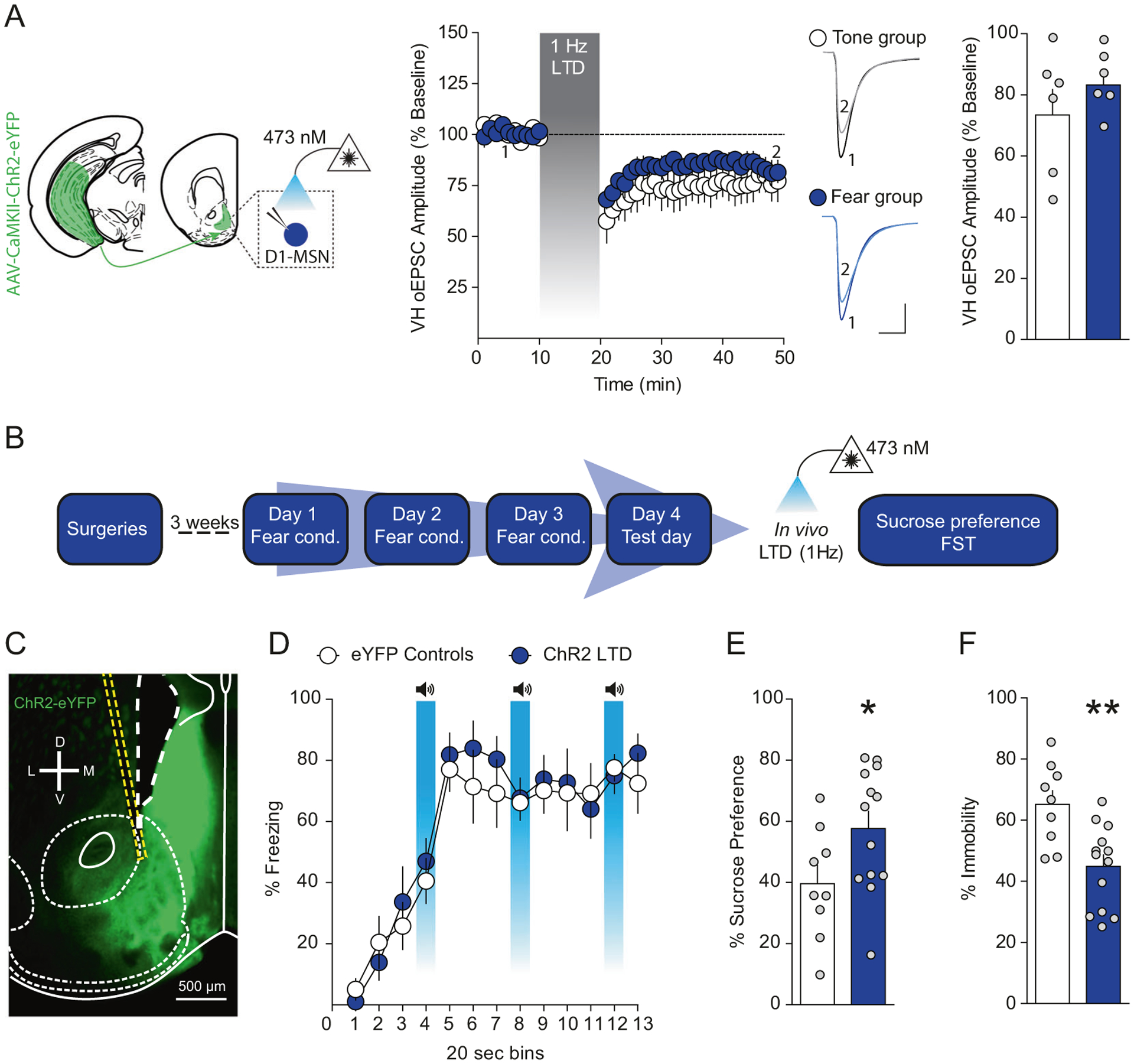
Depressing VH afferents to the NAc rescues anhedonia and passive coping after footshock stress. **a** 1 Hz low-frequency stimulation of VH afferents induces long-term depression (LTD) of VH-oEPSCs in brain slices (*n* = 6 per group; two-way ANOVA; treatment main effect; *F*_(1, 10)_ = 1.1, *p* = 0.32; time main effect; *F*_(38, 380)_ = 13.67, *p* < 0.0001; treatment × time interaction; *F*_(38, 380)_ = 0.9, *p* = 0.64). No difference in the magnitude of LFS-induced LTD of VH synaptic efficacy between control and stressed mice (*n* = 6 per group; *t*-test; *t*_(10)_ = 1.07, *p* = 0.31). **b** Experimental timeline to determine the role of VH inputs to the NAc on affective behavior using in vivo optogenetic low-frequency stimulation. **c** Representative image of NAc ChR2-eYFP expression and optical fiber placement in the NAc. **d** 1 Hz LTD of VH afferents prior to testing does not modify freezing during fear recall (*n* = 10 per group; two-way ANOVA; treatment main effect; *F*_(1, 18)_ = 0.18, *p* = 0.67; time main effect; *F*_(12,216)_ = 32.59, *p* < 0.0001; treatment × time interaction; *F*_(12, 216)_ = 0.49, *p* = 0.92). **e** In vivo depression of VH afferents in the NAc increases sucrose preference relative to control mice (*n* = 9–13 per group; *t*-test; *t*_(20)_ = 2.154, *p* = 0.0436). **f** 1 Hz LTD of the VH to NAc pathway reduces immobility in the FST (*n* = 9–13 per group; *t*-test; *t*_(20)_ = 3.47, *p* = 0.0024).

To determine whether increased VH synaptic strength after repeated footshock stress is causally linked to stress-induced negative affective states, we utilized in vivo optogenetics to depress VH synapses in the NAcmSh (Fig. S4). Using ex vivo slice electrophysiology, we first established that optogenetic low-frequency stimulation (LFS, 1 Hz for 10 min) of VH afferents induces long-term depression (LTD) of VH-oEPSCs in D1-MSNs in the NAcmSh in both tone and stressed mice (Fig. 4a). To determine whether depotentiation of VH inputs to the NAcmSh using LFS would reverse expression of fear conditioning and/or stress-induced anhedonia and passive coping, we injected mice with a virus expressing either a control eYFP fluorophore or ChR2-eYFP into the VH and implanted optical fibers above the NAcmSh (Fig. 4b, c). We applied 1 Hz optogenetic stimulation in vivo prior to the fear test on day 4 in control mice expressing eYFP or mice expressing ChR2-eYFP and observed that 1 Hz LTD of VH inputs to the NAcmSh failed to modify freezing during recall of fear (Fig. S4), suggesting that strengthening of VH inputs to the NAcmSh is not responsible for expression of conditioned fear. In a separate group of mice, we determined whether 1 Hz LTD of VH synapses in the NAcmSh ameliorated anhedonia and passive coping. Both anhedonia and passive coping were still present in eYFP control mice. However, LTD of NAcmSh VH afferents in ChR2-eYFP mice reversed stress-induced anhedonia in the sucrose preference test and passive coping in the forced swim test (Fig. 4e, f). These results indicate that strengthening of VH synapses in the NAcmSh is required for anhedonia and passive coping induced by repeated stress.

### D1-MSN intrinsic excitability is enhanced after stress via decreased IRK currents

**Fig. 5:**
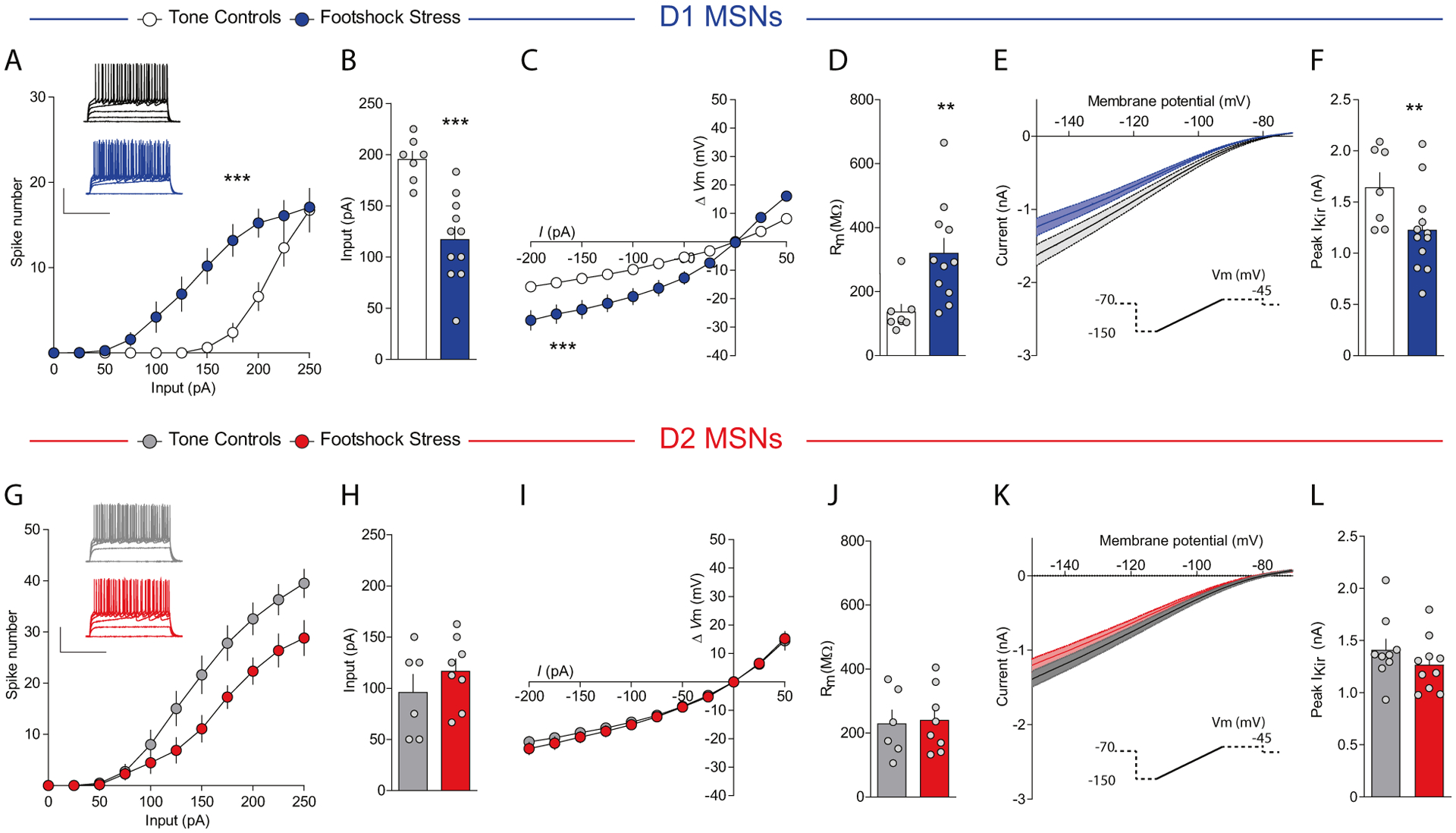
Footshock stress increases intrinsic excitability of D1-MSNs via reduced inwardly rectifying potassium channel activity. **a** Input–output curves of action potential firing in D1-MSNs from control (white) and mice exposed to footshock stress (blue) (*n* = 9–15 per group; two-way ANOVA; treatment × current input interaction; *F*_(10, 220)_ = 5.15, *p* < 0.0001). **b** Rheobase in D1-MSNs (*n* = 7–11; *t*-test; *t*_(16)_ = 4.58, *p* = 0.0003). **c** Current-voltage relationships in D1-MSNs (*n* = 8–12 per group; two-way ANOVA; treatment × current input interaction; *F*_(10, 180)_ = 8.26, *p* < 0.0001). **d** Input resistance in D1-MSNs (*n* = 8–11; *t*-test; *t*_(17)_ = 3.11, *p* = 0.006). **e** Ba^2+^-sensitive IRK currents in D1-MSNs from tone controls and footshock stress mice. **f** Peak IRK currents in D1-MSNs (*n* = 7–12; *t*-test; *t*_(17)_ = 2.28, *p* = 0.036). **g** Input–output curves of action potential firing in D2-MSNs from control (gray) and mice exposed to footshock stress (red) (*n* = 8–10 per group; two-way ANOVA; treatment × current input interaction; *F*_(10, 160)_ = 3.48, *p* = 0.0004). **h** Rheobase in D2-MSNs (*n* = 8–10 per group; *t*-test; *t*_(12)_ = 1.02, *p* = 0.33). **i** Current-voltage relationships in D2-MSNs (*n* = 6–8 per group; two-way ANOVA; treatment × current input interaction; *F*_(10, 120)_ = 0.18, *p* = 0.997; treatment main effect *F*_(1, 12)_ = 0.38, *p* = 0.55; current input main effect *F*_(10, 120)_ = 110.99, *p* < 0.0001). **j** Input resistance in D2-MSNs (*n* = 6–8; *t*-test; *t*_(12)_ = 0.20, *p* = 0.85). **k** IRK currents in D2-MSNs from tone controls and footshock stress mice. **l** Peak IRK currents in D2-MSNs (*n* = 9–10; *t*-test; *t*_(9)_ = 1.085, *p* = 0.2930).

We then studied the effects of footshock stress on intrinsic excitability of D1- and D2-MSNs. Stress increased excitability of NAcmSh D1-MSNs and decreased rheobase, the minimal current amplitude needed to fire an action potential, in these cells relative to controls (Fig. 5a, b). Conversely, D2-MSN excitability was attenuated in stressed mice relative to controls, without an accompanying change in rheobase (Fig. 5g, h). D1-MSNs still displayed increased excitability in the presence of DNQX, AP-V, and picrotoxin (Fig. S5), suggesting that the increased intrinsic excitability in D1-MSNs is not a consequence of alterations in synaptic transmission. Analysis of the passive membrane properties of D1-MSNs revealed that input resistance was increased in cells from the footshock stress group, due to decreased rectification of the *I/V* curve at hyperpolarizing pulses (Fig. 5c, d). This effect was not evident in D2-MSNs (Fig. 5i, j). Inwardly rectifying potassium (IRK) currents mediate voltage rectification in MSNs in response to hyperpolarizing current pulses. To determine whether increased intrinsic excitability was due to decreased IRK currents, we measured IRK currents in stressed and control mice. D1-MSNs from stressed mice had decreased IRK currents relative to the tone group (Fig. 5e, f), an effect that trended in D2-MSNs but did not reach significance. These results demonstrate that repeated footshock stress selectively increases NAcmSh D1-MSN excitability by decreasing IRK currents.

### NAcmSh D1-MSNs are actively recruited in vivo during aversive learning

**Fig. 6:**
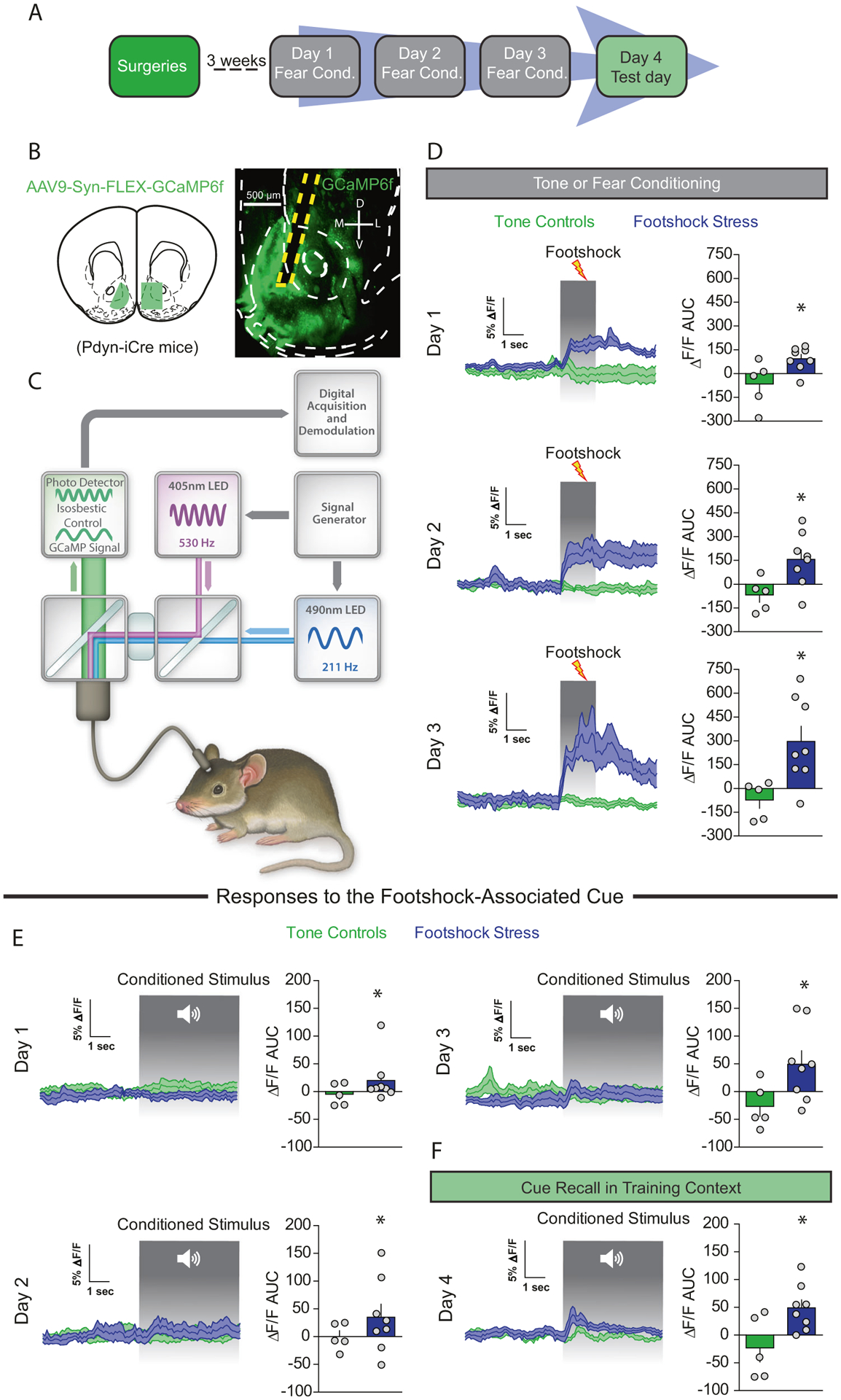
D1-MSNs are recruited by footshock stress and stress-predictive cues. **a** Experimental timeline. In vivo fiber photometry recordings were conducting during conditioning and fear recall procedures (days 1–4). **b** Schematic depicting AAV9-Syn-FLEX-GCaMP6f injection and subsequent optical fiber implantation into the NAc of WT mice. Representative image of optical fiber placement and AAV9-Syn-FLEX-GCaMP6f expression in the NAc of a Dyn-iCre mouse. **c** Schematic of fiber photometry system. **d** Footshock-induced Ca^2+^ activity in D1-MSNs over the course of the 3 day conditioning procedures. Left: time course of D1-MSN bulk Ca^2+^ dynamics during a 5 s window before and after a footshock. Right: area under the curve (AUC) of the Δ*F*/*F*. Day 1 (*t*-test; *t*_(11)_ = 2.62, *p* = 0.024). Day 2 (*t*-test; *t*_(11)_ = 2.66, *p* = *0.02*). AUC day 3 (*t*-test; *t*_(11)_ = 2.86, *p* = 0.016). **e** Cue-induced Ca^2+^ activity during tone or fear conditioning. Left: mean Ca^2+^ Δ*F*/*F* 5 s before and after the onset of the 20 s auditory cue. Right: AUC of the Δ*F*/*F*. AUC day 1 (*t*-test; *t*_(11)_=1.25, *p* = 0.24). AUC day 2 (*t*-test; *t*_(11)_ = 1.13, *p* = 0.28). AUC day 3 (*t*-test; *t*_(11)_ = 2.23, *p* = 0.04). **f** Cue-mediated Ca^2+^ activity during fear recall in the training context. Right: AUC day 4 (*t*-test; *t*_(11)_ = 2.69, *p* = 0.02).

To determine whether footshock stress recruits D1-MSNs in vivo, we monitored D1-MSN Ca^2+^ dynamics during footshock stress in freely moving animals utilizing fiber photometry. To this end, we injected Dynorphin (Dyn)-iCre mice with a Cre-dependent virus expressing GCaMP6f; Dyn is an opioid peptide selectively expressed in D1-MSNs, thus virus expression is restricted to D1-MSNs (Figs. 6a–c and S6). D1-MSNs displayed increases in Ca^2+^ in response to footshocks, but no initial responses to the cue (Fig. 6d). However, with repeated cue-footshock pairings, D1-MSNs developed conditioned Ca^2+^ responses to the footshock-predictive auditory cue (Fig. 6e). On the test day, D1-MSNs from mice in the footshock group displayed conditioned responses to the cue (Fig. 6f). These results suggest that D1-MSNs encode aversive stimuli and display responses to threat-predictive cues, similar to the results obtained for VH terminals in the NAcmSh. These results provide a mechanism by which repeated footshocks recruit D1-MSNs and may potentially lead to increased D1-MSN excitability via reduced IRK currents.

### Increased intrinsic excitability of D1-MSNs drives stress-induced anhedonia and passive coping

**Fig. 7:**
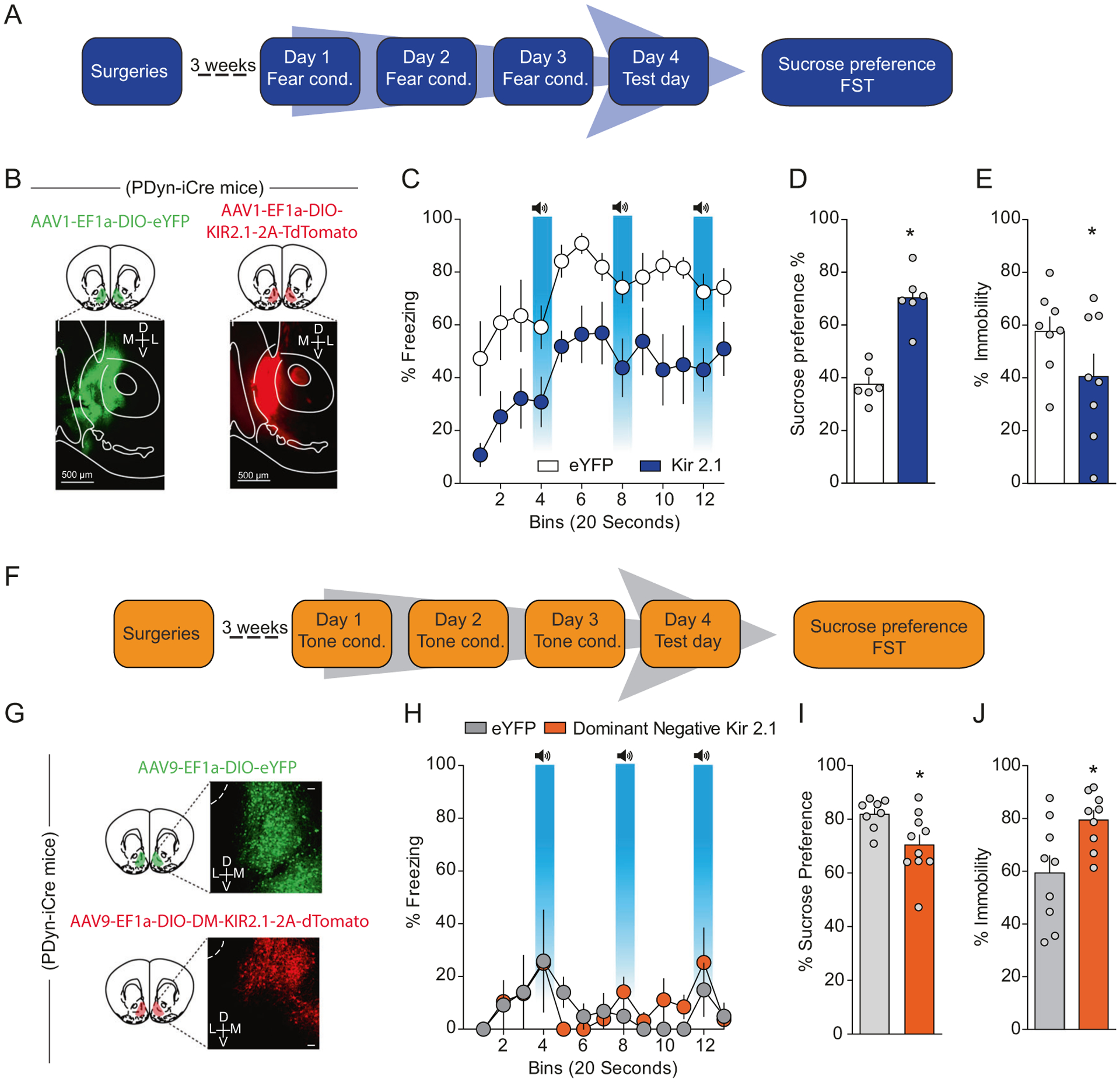
Increased D1-MSN excitability is necessary for stress-induced anhedonia and passive coping, and sufficient to promote negative affect in naïve mice. **a** Experimental timeline. **b** Representative image of AAV_1_-EF1α-DIO-eYFP and AAV_1_-EF1α-DIO-KIR_2.1_-2A-tdtomato expression in Dyn-Cre mice exposed to footshock stress. **c** Freezing to the training context and in response to fear-predictive cues on the day 4 test day. **d** Sucrose preference (*n* = 6; *t*-test; *t*_(10)_ = 6.54, *p* < 0.0001) and (**e**) immobility in the FST across time (two-way ANOVA; treatment × time interaction; *F*_(5, 70)_ = 2.701, *p* = 0.0273) in mice selectively expressing eYFP or KIR2.1 in D1-MSNs (**f**) experimental timeline. **g** Representative image of AAV_9_-EF1α-DIO-eYFP and AAV_9_-EF1α-DIO-KIR_DN_-2A-tdtomato expression in Dyn-Cre mice exposed to footshock stress. **h** Lack of generalized fear in mice expressing KIR DN in D1-MSNs. **i** Sucrose preference in naive mice selectively expressing eYFP or KIR_DN_ in D1-MSNs (*n* = 9; *t*-test; *t*_(16)_ = 2.53, *p* = 0.022). **j** Time spent immobile in naive mice selectively expressing eYFP or KIR_DN_ in D1-MSNs (*n* = 9; *t*-test; *t*_(16)_ = 2.707, *p* = 0.016).

Next, we asked whether the observed increased D1-MSN excitability via decreased IRK function mediates negative affect after repeated footshock stress. To this end, we overexpressed a modified IRK channel, *Kir2.1* [27], in a Cre-dependent manner in Dyn-iCre mice (Fig. 7a, b). Overexpression of KIR2.1 in D1-MSNs resulted in increased IRK currents, decreased input resistance, and decreased intrinsic excitability (Fig. S7A–C), demonstrating that this viral approach results in functional decreases of D1-MSN excitability. Overexpression of *Kir2.1* in D1-MSNs decreased freezing to the context and cues associated with footshocks relative to mice expressing a control fluorophore on day 4 (Fig. 7c), suggesting that D1-MSN hyperexcitability promotes conditioned fear. Importantly, decreased freezing was not mediated by changes in locomotor activity or anxiety as locomotor activity and anxiety-like behaviors were not modified in separate Dyn-iCre mice expressing *Kir2.1* in NAcmSh D1-MSNs (Fig. S8). We subsequently determined whether selective viral-mediated *Kir2.1* expression in D1-MSNs was able to rescue stress-induced anhedonia and passive coping. Both decreased sucrose preference and increased immobility in the forced swim test were present in stressed mice expressing a control fluorophore (Fig. 7d, e). However, *Kir2.1* overexpression reversed stress-induced anhedonia as assessed by the sucrose preference test, as well as stress-induced passive coping in the forced swim test (Fig. 7d, e). Collectively, these results demonstrate that decreased IRK currents in D1-MSNs lead to increased D1-MSN excitability and that increased D1-MSN intrinsic excitability promotes the development of negative affective states after repeated stressful experiences. Similar findings were also observed in an animal model of inflammatory pain [28] and after chronic social defeat stress [20].

### Enhancing D1-MSN intrinsic excitability via decreased IRK currents drives anhedonia and passive coping in naïve mice

Next, we determined whether a reduction in IRK currents in D1-MSNs was able to drive anhedonia and passive coping. To this end, we injected a Cre-dependent virus into the NAcmSh of Dyn-iCre mice expressing a mutant-pore KIR2.1 [27] that functions as a dominant-negative *Kir2.1* (Figs. 7g and S7). D1-MSNs expressing a dominant-negative *Kir2.1* displayed decreased IRK currents, increased input resistance, and increased intrinsic excitability (Fig. S7D–F), confirming that this virus is efficiently interfering with the function of endogenous KIR channels. To establish whether increased excitability of NAcmSh D1-MSNs was sufficient to drive anhedonia and passive coping, mice expressing the dominant-negative *Kir2.1* were exposed to the auditory stimuli without the footshocks (Fig. 7f, h) as in the control group and subsequently tested on sucrose preference and forced swim procedures. Decreasing IRK currents with a dominant-negative *Kir2.1* in D1-MSNs produced a significant decrease in sucrose preference and increased immobility in the forced swim test relative to control mice (Fig. 7i, j). Collectively, these results demonstrate that increased D1-MSN activity via decreased IRK currents is capable of driving anhedonia and passive coping in naïve mice, and necessary for stress-induced anhedonia and passive coping as a consequence of repeated footshock stress.

### Synaptic and intrinsic plasticity onto D1-MSN co-participate in stress-induced anhedonia and passive coping

**Fig. 8:**
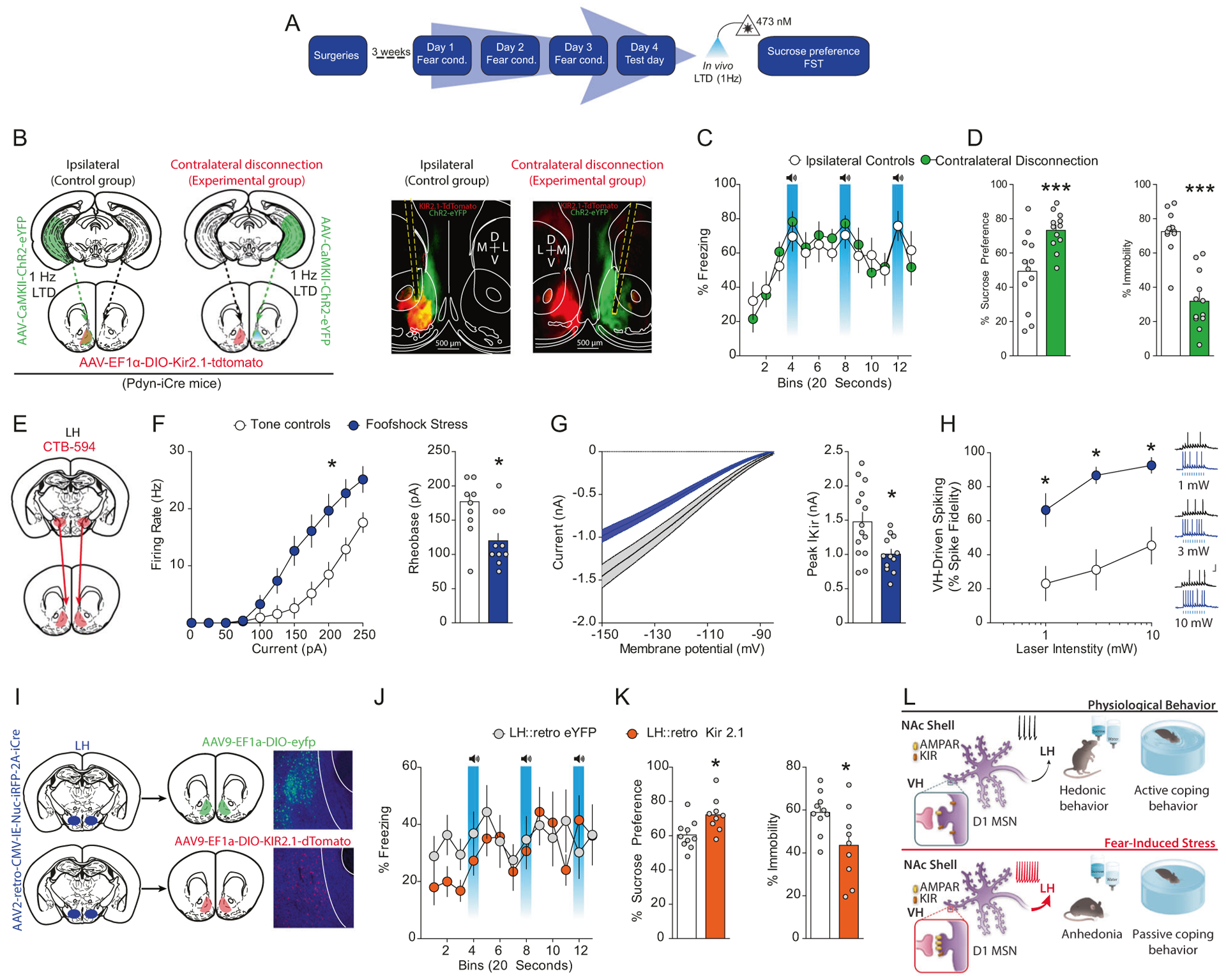
Cooperation of synaptic and intrinsic plasticity in a disynaptic circuit drives stress-induced anhedonia and passive coping. **a** Experimental timeline. **b** Mice were injected with unilateral AAV_1_-CaMKII-ChR2-eYFP into the VH. Contralateral disconnection and ipsilateral control group mice were injected with AAV_1_-EF1α-DIO-KIR_2.1_-2A-tdtomato into the contralateral or ipsilateral NAc, respectively. **c** Freezing on day 4 was not significantly different between control and footshock stress mice (*n* = 11–12 per group; two-way ANOVA; treatment main effect; *F*_(1,21)_ = 0.01, *p* = 0.91; time main effect; *F*_(14,294)_ = 8.83, *p* < 0.001; treatment × time interaction; *F*_(14, 294)_ = 0.94, *p* = 0.52). **d** Contralateral disconnection of increased VH synaptic strength in the NAc and increased excitability of NAc D1-MSNs restores sucrose preference (*n* = 12; *t*-test; *t*_(22)_ = 3.36, *p* = 0.003) and mobility in the forced swim test (*n* = 11–12; *t*-test; *t*_(21)_ = 6.42, *p* < 0.0001). **e** CTB-594 was injected into the LH of WT mice. LH-projecting D1-MSNs were recorded. **f** Input–output curves and rheobase of action potential firing in LH-projecting NAc D1-MSNs. Input–output curves (*n* = 12–13 per group; two-way ANOVA; treatment × current interaction; *F*_(10, 230)_ = 8.92, *p* < 0.0001). Rheobase (*t*-test; *t*_(23)_ = 3.77, *p* = 0.001). **g** Decreased IRK currents in LH-projecting D1-MSNs from stressed mice (*t*-test; *t*_(24)_ = 2.84, *p* = 0.0091). **h** AAV_1_-CaMKII-ChR2-eYFP and CTB-594 were injected into the VH and LH, respectively, of mice. LH-projecting NAc D1-MSNs were recorded. Increased input–output transformations in the VH-NAc D1-MSN-LH pathway in naïve and footshock stress mice (two-way ANOVA; treatment main effect; *F*_(1, 13)_ = 29.84, *p* = 0.0001; stimulation intensity main effect; *F*_(2, 26)_ = 16.11, *p* < 0.0001; treatment × stimulation intensity interaction; *F*_(2, 26)_ = 1.53, *p* = 0.236). **i** AAV_2_-retro-CMV-iRFP-2A-Cre was injected into the LH, while AAV_9_-EF1a-DIO-KIR2.1–2A-tdtomato or AAV_9_-EF1a-DIO-eYFP was injected into the NAc of WT mice. **j** Freezing on day 4 in mice expressing control fluorophore or KIR2.1-tdtomato in LH-projecting D1-MSNs. **k** Overexpression of KIR2.1 in LH-projecting D1-MSNs rescues footshock stress-induced decreases in sucrose preference (*n* = 9–10; *t*-test; *t*_(17)_ = 2.80, *p* = 0.012) and increases in immobility in the forced swim test (*n* = 9–10; *t*-test; *t*_(17)_ = 2.417, *p* = *0*.0*27*). **l** Model depicting that increased information flow in a VH to NAc to LH circuit drives passive coping and anhedonia. This is mediated by increased synaptic strength of VH inputs to D1-MSNs, via increased AMPAR function and or number, and increased excitability, via decreased IRK function and/or number, in LH.

Are synaptic plasticity of VH afferents and intrinsic plasticity in D1-MSNs factors that co-participate in the expression of anhedonia and passive coping after stress? To address this question, we performed a novel molecular/optogenetic disconnection procedure to determine whether synaptic and intrinsic adaptations in D1-MSNs co-participate to drive anhedonia and passive coping (Fig. 8a, b). In these experiments, control Dyn-iCre mice were injected with unilateral AAV_1_-EF1α-DIO-KIR2.1–2A-tdtomato into the NAcmSh and AAV_1_-CaMKII-ChR2-eYFP into the ipsilateral VH. These mice also had an optical fiber implanted into the NAcmSh ipsilateral to the AAV_1_-CaM-KII-ChR2-eYFP VH injection to induce LTD of the VH hippocampus to NAcmSh pathway. In the control ipsilateral group, the contralateral, unmanipulated hemisphere is still capable of processing information, and hence supporting behavior. Experimental Dyn-iCre mice undergoing molecular/optogenetic disconnections were injected with unilateral AAV_1_-EF1α-DIO-KIR2.1–2A-tdtomato into the NAc, while an optical fiber was implanted into the contralateral NAcmSh and AAV_1_-CaMKII-ChR2-eYFP was injected into the VH ipsilateral to the NAcmSh optical fiber to induce LTD of VH to NAcmSh afferents. If anhedonia and passive coping induced by stress do not rely on both VH synaptic plasticity and hyperexcitability in D1-MSNs co-participation, then independently manipulating each of these processes in each hemisphere should not bilaterally disrupt the NAc mechanisms driving negative affect. However, if stress-induced negative affect is mediated by concomitant strengthening of VH synapses onto D1-MSNs and increased excitability of D1-MSNs, then molecular/optogenetic disconnection of these processes should result in bilateral disruption of VH to NAc D1-MSN information flow. Contralateral disconnection of VH potentiation of synaptic transmission into the NAc and D1-MSN hyperexcitability increased sucrose preference relative to ipsilateral control mice (Fig. 8d). Furthermore, immobility in the forced swim test was decreased in contralateral disconnection mice relative to controls (Fig. 8d). Taken together, these findings are consistent with the hypothesis that increased information flow via strengthening of VH inputs to D1-MSNs and increased excitability of D1-MSNs drives anhedonia and passive coping.

### Overexpressing *Kir2.1* in D1-MSNs projecting to LH rescues stress-induced anhedonia and passive coping

D1-MSNs project to a variety of downstream targets, including the LH, which has been implicated in mediating hedonic states and motivation [29]. NAc D1-MSNs are the predominant NAc cell type (~95%) that innervate the LH [30–32]. Moreover, LH-projecting D1-MSNs do not strongly collateralize to the midbrain, suggesting that the NAcmSh to LH represents a separate pathway from the NAcmSh to VTA pathway [30–32]. We first determined whether accumbal neurons projecting to the LH underwent plasticity of intrinsic excitability. To this end, we injected mice with cholera toxin subunit B conjugated to Alexa fluor 594 (CTB-594) into the LH, a means to retrogradely label NAcmSh MSNs projecting to the LH (putatively D1-MSNs), and performed whole-cell recordings from LH-projecting MSNs in both tone control and footshock-stressed mice (Fig. 8e). LH-projecting NAc MSNs from footshock-stressed mice had increased intrinsic excitability and a decrease in IRK channel currents, similar to the previous recordings from D1-TdTomato mice (Fig. 8f, g). We subsequently determined whether stress-induced potentiation of VH inputs and increased intrinsic excitability in LH-projecting D1-MSNs result in increased input/output transformations in the VH → NAc D1-MSN → LH circuit, and hence, increased information flow in this disynaptic circuit. We injected mice with CTB-594 in the LH and AAV_1_^−^CaMKII-ChR2-eYFP into the VH. Using whole-cell electrophysiology, we subsequently determined whether footshock stress increased the ability of VH inputs to drive action potentials in LH-projecting D1-MSNs, that is increasing input/output transformations in the VH → NAc D1-MSN → LH disynaptic circuit. We employed optogenetic train stimulation of ChR2-containing VH afferents to drive postsynaptic firing in NAc CTB-594-positive D1-MSNs and measured excitatory drive of action potential firing in stressed and control mice. Train stimulation of VH afferents resulted in enhanced action potential firing in stressed mice relative to their control counterparts (Fig. 8h). These results suggest that synaptic and intrinsic plasticity cooperate to increase information flow in a VH → NAc D1-MSN → LH circuit.

To determine whether decreased IRK currents in LH-projecting NAc D1-MSNs-mediated stress-induced anhedonia and passive coping, we selectively overexpressed *Kir2.1* in LH-projecting D1-MSNs by injecting mice with AAV_2_-retro-CMV-iRFP-2A-Cre into the LH and either AAV_9_-EF1α-DIO-KIR2.1–2A-tdtomato or AAV_9_-EF1α-DIO-eYFP into the NAc (Fig. 8i). Both groups of mice underwent footshock stress (Fig. 8j) and were subsequently tested in the sucrose preference and forced swim test. eYFP-expressing control mice had reduced sucrose preference and increased immobility in the forced swim test relative to mice expressing *Kir2.1* in LH-projecting NAc D1-MSNs (Fig. 8k). These results suggest that reduced KIR-mediated IRK currents in LH-projecting D1-MSNs enhances their excitability and that the increased output from the NAc to the LH drives stress-induced anhedonia and passive coping.

## Discussion

Our findings identify a disynaptic limbic circuit that underlies stress-induced anhedonia- and passive coping-like behavioral abnormalities in mice (Fig. 8l). We show that stress recruits VH afferents to the NAc and selectively potentiates VH synapses onto NAcmSh D1-MSNs. Depotentiation of VH inputs to the NAc rescues stress-induced anhedonia and passive coping. In addition, stress increases intrinsic excitability of LH-projecting D1-MSNs via reduction in IRK currents. Restoring KIR channels in D1-MSNs ameliorates stress-induced anhedonia and passive coping. On the other hand, artificially decreasing IRK currents in D1-MSNs is sufficient to drive anhedonia and passive coping in naïve mice. Finally, by employing a novel contralateral disconnection strategy with input and cell-type specificity, we demonstrate that stress-induced maladaptive behavior is casually related to increased information flow in a VH → NAc D1-MSN → LH circuit and requires co-participation of synaptic and intrinsic plasticity onto D1-MSNs (Fig. 8l). Thus, this study provides the first demonstration that both synaptic and intrinsic plasticity cooperate to change input–output activity in a distributed limbic circuit to promote stress-induced maladaptive behavior.

### Potentiated VH → NAcmSh D1-MSN activity defines stress-induced anhedonia and passive coping

Previous studies report a crucial role of the VH formation overall in mediating negative affective states triggered by stress [26] as well as behavioral inhibition in the context of goal directed behaviors [25]. The first demonstration by Bagot et al. [26] that VH afferents to the NAc promote depressive-like behavior induced by stress is grounded in a vast literature. Synchronization of VH activity with other limbic structures has been described in the context of depressive-like behavior and outputs from the VH have been shown to mediate different aspects of negative affect and decreased motivational states [33]. For example, VH outputs to prefrontal cortex and LH are critical for anxiety-like behavior [34, 35], while VH activity to the septum is sufficient to decrease consummatory behavior [36]. Here, we show that VH outputs to the NAc are actively recruited during stress and potentiated onto D1-MSNs, and this potentiated VH → NAc D1-MSN activity is causally linked to stress-induced anhedonia and passive coping. We also provide evidence that the VH to NAc pathway does not carry information that is relevant for the expression of learned fear but rather about the emotional status dictated by exposure to stress. Taken together, these findings elucidate how the hippocampal formation may encode and represent different aspects of an aversive experience, by engaging specific downstream targets. The hippocampal formation is indeed a functionally and anatomically heterogeneous entity [37]. In support of this, projection and expression patterns of genes starkly differ between the dorsal and VH, as well as the roles of those axes in emotional behavior [38, 39], with the VH playing a crucial role in mediating negative affect. For instance, silencing the ventral dentate gyrus with either inhibitory chemogenetic or viral strategies that control glutamate homeostasis and limit glutamatergic synaptic transmission confers resilience to stress [40, 41], while overexpression of stress susceptibility hub genes (Dkkl1 and Sdk1) that promote negative affect increases excitatory drive onto VH pyramidal neurons [26]. Moreover, overexpression of Sdk1 within the VH changes brain-wide network activity similar to other established stress models [33], suggesting that VH hyperactivity may be a common feature that drives stress-induced changes in network function that mediate negative affect.

Our results are also in agreement with previous findings showing that adverse experiences, such as chronic stress or pain, increase synaptic transmission at VH to NAc synapses [26, 42]. Other studies have demonstrated that depotentiation or optogenetic stimulation of VH terminals within the NAc prevents stress-induced social interaction deficits and is sufficient to drive passive coping in naïve animals, respectively [26]. Furthermore, decreased VH activity in the NAc is observed at the onset of consummatory behavior, and inhibition of VH to NAc synaptic transmission promotes feeding [43]. Collectively, these results provide a framework in which stress increases synaptic transmission from the VH to the NAc, which in turn drives anhedonia and decreases active coping strategies. It should be noted that in the present study negative affect was indexed by a decrease in sucrose preference and increase in immobility in the forced swim test. These tests are not exhaustive in describing maladaptive negative affect, though attempts were made to determine if the footshock stress-induced maladaptive behavior included anxiety-like behavior. Nonetheless, the disynaptic limbic circuit from the VH to NAcc to LH described here is pertinent to maladaptive negative affect relevant to a plethora of psychiatric disorders, including drug addiction [44]. Addictive behaviors and relapse are in part driven by negative reinforcement [45], which is drug-seeking behavior aimed at alleviating negative affective states, craving and/or stress. Consistent with the hypothesis that the VH to NAc pathway is relevant for driving negative affective states that promote maladaptive behavior, interrupting information processing between the VH and NAc decreases relapse in animals that underwent punishment-induced abstinence and in drug withdrawn animals [46–49].

Other studies provide evidence that the VH to NAc pathway promotes motivated behaviors of positive valence. For example, VH to NAc pathway supports social memory [50, 51] and mice will lever press for optogenetic self-stimulation of the VH to NAc pathway [8]. A recent study demonstrated that high frequency (100 Hz) optogenetic stimulation of VH to NAc using canonical ChR2 is capable of producing a conditioned place preference [50]. Moreover, chronic multimodal stress involving concomitant restraint stress and noxious auditory stimuli decreases synaptic efficacy from the VH to D1-MSNs [50]. A number of methodological issues may explain the discrepant findings. For instance, ChR2 kinetics do not allow for high fidelity responding at 100 Hz (ChR2 fidelity substantially drops off at 40 Hz). It is possible that 100 Hz optogenetic stimulation may be effectively shutting down the VH to NAc pathway. Moreover, differential targeting of the hippocampal formation and the NAcc (ventral subiculum and NAc shell in the present study vs. dorsal aspects of the hippocampus and more lateral NAc in the LeGates et al. study) might account for discrepancies between these studies. Nonetheless, the overwhelming literature supports a role for excessive VH formation activity in driving negative affect and reward deficits, and this is at least partially driven by VH inputs to the NAc. Therefore, in addition to the role of the VH to NAc pathway in processing negative motivational valence, this pathway might also play a role in mediating positive motivational valence.

### Enhanced D1-MSN → LH intrinsic excitability defines stress-induced anhedonia and passive coping

D1-MSNs project to multiple downstream targets including the midbrain, LH, and VP. D1-MSNs are differentially localized within the NAc subcompartments depending on their projection target. For instance, LH-projecting MSNs are preferentially localized in the medial aspect of the NAc shell, while midbrain-projecting D1-MSNs are concentrated in the NAc core [30, 31, 52, 53]. Furthermore, D1-MSNs constitute more than 95% of the NAc neurons that project to the LH [30–32], with minimal overlap in LH- and VTA-projecting D1-MSNs [30, 32]. Global activation and inhibition of the NAc shell suppresses and increases feeding, respectively [54–56], and NAc shell control of feeding is mediated by the LH [31, 57, 58]. Selective optogenetic activation of D1-MSNs that project to the LH suppresses feeding and promotes passive coping [31, 59], and facilitates extinction of reward-seeking behavior [32]. In line with those results, we find that stress increases LH-projecting D1-MSN excitability via decreased IRK function, and that reversing the observed hyperexcitability in LH-projecting D1-MSNs rescues stress-induced anhedonia and passive coping. Thus, these results provide a physiological framework wherein stress increases LH-projecting D1-MSN activity via decreased IRK currents, which in turn suppresses LH activity and leads to a reduced drive to achieve a physiological hedonic state and interferes with active coping mechanisms. Thus, in addition to playing a major role in orchestrating appetitive motivated behavior, D1-MSNs in the NAc shell also play a major role in mediating stress-induced maladaptive behavior in large part through outputs to the LH.

The importance of K^+^ channels as therapeutic targets in the context of mood disorders is rapidly emerging [60, 61]. In a recent clinical study, the KCNQ-selective channel potentiator ezogabine was shown to have an antidepressant effect in subjects with MDD [62]. Interestingly, ezogabine administration is associated with an improvement in depressive and anhedonic symptoms together with decreased functional connectivity between the ventral caudate, midcingulate cortex, and posterior cingulate cortex [62]. These results suggest that the antidepressant action of ezogabine may in part be mediated by NAc circuitry, presumably by decreasing excessive activity within this structure.

### Engagement of synergistic synaptic and intrinsic plasticity in stress-induced maladaptive behavior

Our data suggest that stress induces maladaptive excitation-spike coupling in a disynaptic limbic circuit from VH → NAc D1-MSN → LH, representing a major pathological condition that “tonically” limits the expression of physiologically and behaviorally relevant hedonic states and the execution of active coping strategies. Importantly, this body of work provides the first demonstration that exposure to stress engages cooperative synaptic and intrinsic excitability plasticity to shape behaviorally relevant circuit activity. In addition, this study helps clarify how extrapolating global changes in neuronal excitability solely based on changes in synaptic strength, without considering nonsynaptic factors (“intrinsic” membrane properties), does not provide a complete readout of how behaviorally relevant experiences alter general neuronal activity. In fact, intrinsic factors are pivotal for determining the probability that a neuron will fire an action potential in response to incoming excitatory synaptic inputs [63]. Moreover, intrinsic excitability govern the rules for induction of synaptic plasticity and, conversely, intrinsic factors are themselves subjected to regulation by synaptic inputs and patterns of synaptic activity [63]. Stress-induced downregulation in D1-MSN dendritic Kir channel number or function may enhance all electronic signals (inhibitory or excitatory) in cells, essentially increasing the gain of the cell, or specifically interact with synapses potentiated by stress, such as the VH. Kir channels are also localized at asymmetric synapses in addition to dendrites [64], raising the possibility that Kir may be able to filter synapse-specific activity. Moreover, Kir channels are inactivated with depolarizing potentials, therefore it is possible that potentiated VH synapses would more effectively inactivate Kir channels accumulated in or around the VH synapse, facilitating synaptic integration [65, 66]. Lastly, with repeated chronic social defeat stress, pruning of excitatory synapses in D1-MSNs has been reported [20, 67]. Thus, it is possible that within NAcmsh D1-MSNs, a subset of asymmetric synapses not innervated by the VH input are selectively pruned, a putative homeostatic mechanism to compensate for overdrive exerted by the VH. Thus, even though a downregulation of IRKs may act to increase the gain of all electrical signals onto D1-MSNs, potentiation of VH afferents in the face of reductions in Kir-mediated currents and changes in general excitatory drive onto D1-MSNs is likely to increase the signal-to-noise ratio at this pathway. The mechanism by which stress affects VH plasticity selectively onto hyperexcitable NAc D1-MSNs is still unclear. Moreover, it remains unknown whether D1-MSN hyperexcitability appears before, after, or concomitant with synaptic plasticity at VH glutamatergic synapses. Therefore, future work addressing those points is necessary.

Future studies are also needed for answering several additional questions: does the interplay between intrinsic and synaptic plasticity represents a positive or a negative feedback mechanism for neuronal function in other neuropsychiatric conditions? Does this regulate the long-term stability of synaptic weight, neuronal input–output relationships, and network dynamics? Moreover, is this interplay suitable for modulation to exert therapeutic intervention? For example, understanding whether and how *Kir2.1* channel function or VH activity respond to currently available medications, cognitive or surgical (i.e., deep brain stimulation) therapies may direct discovery efforts toward resilience-enhancing strategies for therapeutic intervention. A suitable candidate may be repetitive transcranial magnetic stimulation [68]. This innovative treatment influences neural activity by acting on neuroplasticity both locally, under the stimulation site, and importantly it may affect synaptic transmission within a distributed neural network, including the NAc. Interestingly, preclinical literature suggests that the NAc, a deep brain region, may also be suited for therapeutic purposes via noninvasive deep brain stimulation [69].

It is increasingly clear that distributed networks mediate fluctuations in mood, as well as susceptibility and resilience to stressors drive negative affect. Fluctuations in mood have been attributed to functional changes in several cortical, basal ganglia, and limbic circuits [33, 70–72], including the extended amygdala and lateral habenula [73, 74]. Of interest, reversing or mimicking adaptations in several regions is necessary and sufficient to promote negative affect and in regulating the intensity and initiation of the emotional response. Thus, brain-wide changes in information processing may be critical for the full expression of maladaptive behavior, as it has been previously demonstrated [33]. This is in agreement with the present study elucidating a disynaptic circuit in mediating stress-induced negative affect. This is of relevance as it suggests that therapeutic treatments could be aimed at restoring network function downstream of sites where plasticity has occurred.

In conclusion, our findings demonstrate that mitigating VH over-activation and hyperexcitability of LH-projecting NAc D1-MSNs may represent a circuit- and cellular-based therapeutic intervention for neuropsychiatric conditions where anhedonia and passive coping are core symptoms.

## Methods

### Subjects

Animals: both male and female mice were used in the study. Transgenic mouse lines used were *ROSA26*^*fstdTomato/*+^ [75], Drd1a-tdtomato [76], and Dyn-IRES-Cre [30, 77]. Animals were housed in temperature- and humidity-controlled facilities under a reverse 12 h light/dark cycle (lights off at 8 a.m.) with ad libitum chow and water. Procedures were conducted during the animals’ dark cycle. Mice were 2–3 months of age at the start of the experiment. Experiments were conducted in accordance with the USPHP *Guide for the Care and Use of Laboratory Animals*, and approved by the Animal Care and Use Committee of the National Institute on Drug Abuse Intramural Research Program.

### Stereotaxic virus injections and optical fiber implantations

Mice were anaesthetized with ketamine (100 mg/kg; ip) and xylazine (10 mg/kg; ip) and subsequently secured to a stereotaxic frame. After exposing the top of the skull, the mouse’s head was leveled by ensuring that the distance in the dorsal/ventral axis between bregma and lambda was within 50 μm. Virus or tracers were injected into the VH (0.5 μl; VH; AP: −3.5; ML: ±3.05; DV: −4.95), NAc (0.3–0.4 μl; NAc; AP: 1.3; ML: ±1.65; DV: −4.5–4.55; 12° angle), LH (0.3 μl LH; AP: −1.2; ML: ±1.05; DV: −5.5), or VTA (0.3 μl VTA; AP: −3.2; ML: ±1.6; DV: −4.65; 14° angle) with 29 Ga stainless steel tubing connected to FEP tubing. Virus was injected over 5 min utilizing a 2 μl Hamilton syringe. Injectors were left in place for 8 additional minutes before slowly retracting the injector. Incisions were subsequently stapled. Animals recovered on a warm heating pad before being transferred back to the vivarium home cage. Electrophysiological experiments were conducted 4–6 weeks after virus injection into the VH, 3–4 weeks after injection into the NAc, and 6–7 days after CTB injection. Behavior experiments were conducted starting 3–4 weeks after virus injection.

For in vivo optogenetic experiments, mice were injected with virus into the VH (0.5 μl). Three to four weeks later, 200 μm core optical fibers threaded through 1.25 mm zirconia ferrules were implanted into the NAc (AP: 1.3; ML: ±1.65; DV: −4.0; 12° angle). For fiber photometry experiments determining Ca^2+^ dynamics of VH afferents in the NAc, virus expressing GCaMP6s was injected into the VH and 3–4 weeks later a 400 μm core optical fibers threaded through a 1.25 mm zirconia ferrule was implanted into the NAc (AP: + 1.35; ML: ±1.7; DV: −3.7). Behavioral experiments began ~7 days post surgery. For fiber photometry recordings of D1-MSNs, Cre-dependent virus expressing GCaMP6f was injected into the NAc and a 400 μm core optical fiber was implanted into the NAc (AP: +1.35; ML: ±1.7; DV: −3.2).

### Repeated footshock stress

Mice were exposed to repeated footshock stress over the course of 3 days [78]. On day 1, mice were placed in sound-attenuating Med Associates chambers and exposed to four auditory cues (90 dB white noise; 20 s duration) that co-terminated with a mild footshock (2 s duration; 0.6 mA). Cue-footshock pairings were presented on a random interval. Control mice (Tone Group) were presented auditory cues without the footshock. On days 2 and 3, mice were placed in the conditioning chambers and exposed to four cue-footshock pairings similar to day 1. On day 4, the recall of the contextual and cue-associated fear memory was determined by placing mice back in the chambers and presenting conditioned white noise cue three times (20 s duration; 60 s ITI). Freezing was analyzed using Med Associates Video Freeze Software [79]. Similar conditioning procedures were utilized for fiber photometry experiments, with the exception that Coulbourne Instruments chambers were utilized.

### Sucrose preference test

Mice were placed in standard rodent cages (45 × 27 × 15 cm) and given access to a bottle containing tap water and a bottle containing 1% sucrose for 4 h. The total amount of tap water and sucrose consumed in the 4-h period was recorded. Sucrose preference testing occurred ~1–2 h after the start of the animal’s active cycle. Sucrose preference is expressed as the amount of sucrose consumed divided by the total amount of water consumed multiplied by 100.

### Forced swim stress

Mice were placed in glass cylinders (height 30 cm, 20 cm diameter) containing 25 °C water filled up to 15 cm for 6 min. The first 2 min of the forced swim test were not analyzed. Immobility was recorded offline by an observer blind to experimental conditions. The operational definition for mobility in the FST is any movements other than those necessary to balance the body and keep the head above the water.

### Open field maze

Open field maze procedures were conducted as previously described [72]. The open field (45 × 45 cm) was divided into a central field (center, 15 × 15 cm) and an outer field (periphery). Mice were placed in the open field for 10 min and the amount of time spent in the center and periphery of the arena and locomotor activity was recorded. Room lighting was measured with the aid of a lux meter during testing (~200 Lux).

### Light–dark box

The light–dark box consisted of an open compartment (28.7 × 30 × 20.6 cm) exposed to ambient light and an enclosed dark compartment (28.7 × 15 × 20.6 cm). The two compartments were connected via a rectangular door (7 × 3.5 cm). During testing, mice were placed in the light-exposed side of the light–dark box and allowed to explore the arena for 5 min. The latency to enter the dark compartment was recorded offline by an observer blind to experimental conditions. Room lighting was measured with the aid of a lux meter during testing (~200 Lux).

### Elevated plus maze

Elevated maze procedures were conducted as previously described [72]. Mice were placed on an elevated plus maze (30 cm elevation) consisting of two open and two open arms for 5 min. The amount of time spent in the open and closed arms was recorded utilizing Ethovision XT tracking software. The following index was used: (time spent in the open arms/(time spent in open arms + time spent in closed arms)) × 100. Room lighting was measured with the aid of a lux meter during testing (~200 Lux).

### Fiber photometry

For all recordings, GCaMP6 was excited at two wavelengths (490 nm, calcium-dependent signal and 405 nm isosbestic control; [80]) by amplitude modulated signals from two light-emitting diodes reflected off dichroic mirrors and coupled into a 400 μm 0.48NA optic fiber. Signals emitted from GCaMP6s and its isosbestic control channel then returned through the same optic fiber, and were acquired using a femtowatt photoreceiver (Model 2151; Newport), digitized at 1 kHz, and then recorded by a real-time signal processor (RZ5D; Tucker Davis Technologies) running the Synapse software suite. Analysis of the resulting signal was then performed using custom-written MATLAB scripts. Changes in fluorescence across the experimental session (Δ*F*/*F*) were calculated by smoothing signals from the isosbestic control channel [80], scaling the isosbestic control signal by regressing it on the smoothed GCaMP signal, and then generating a predicted 405 nm signal using the linear model generated during the regression. Calcium independent signals on the predicted 405 nm channel were then subtracted from the raw GCAMP signal to remove movement, photo bleaching, and fiber bending artifacts. Signals from the GCaMP channel were then divided by the control signal to generate the Δ*F*/*F*. Peri-event histograms were then created by normalizing (robust *z*-scores) the fluorescence to each pre-event baseline period and then averaging changes in normalized fluorescence (Δ*F*/*F*) across repeated trials during windows encompassing behavioral events of interest.

### In vivo optogenetics

For in vivo long-term depression of VH afferents to the NAc, mice were connected to 200 μm core branching patch cords (Doric Lenses) connected to a 473 nm laser (Opto Engine LLC). Low-frequency optogenetic stimulation (1 Hz; 1 ms; 10 mW) was applied to VH afferents in the NAc for 10 min in clean, standard mice housing chambers, ~0–60 min after the tone recall (day 4) of fear conditioning. LTD induction occurred only at this time point, then animals were tested during the following days in the sucrose preference test and FST. For disconnection procedures, unilateral low-frequency stimulation was applied either ipsilateral or contralateral to the NAc injected with KIR2.1-expressing virus, ~30–60 min after the tone recall (day 4) of fear conditioning similar to procedures described above. LTD induction occurred only at this time point, then animals were tested during the following days in the sucrose preference test and FST. After stimulation, mice were subsequently disconnected and returned to their home cages. For the experiment aimed at determining whether depotentiation of VH inputs to the NAcmSh would reverse expression of fear conditioning, we applied 1 Hz optogenetic stimulation in vivo 1 h after day 3 of our fear conditioning protocol, that is ~24 h prior to the fear test on day 4.

### Whole-cell electrophysiology

#### Slice preparation

Slice electrophysiology experiments were carried out as previously described [30, 72]. Mice were anaesthetized with euthasol (Butler-Schein) before decapitation. Brains were rapidly removed and placed in ice-cold NMDG-based cutting solution [81] containing (in mM): 92 NMDG, 20 HEPES, 25 glucose, 30 NaHCO_3_, 2.5 KCl, 1.2 NaPO_4_, 5 sodium ascorbate, 3 sodium pyruvate, and 2 thiourea saturated with 95% O_2_/5% CO_2_ with an osmolarity of 303–306 mOsm (Advanced Cell Diagnostics). The frontal lobe was rapidly blocked, dried on filter paper, and glued to a Leica VT1200 Vibratome platform containing ice-cold NMDG-based cutting solution. Coronal sections (300 μm) containing the NAc were obtained at a speed of 0.07 mm/s. Slices were subsequently incubated in a holding chamber bubbled with NMDG-based cutting solution for 5–10 min at 34 °C. Slices were then transferred to modified holding aCSF saturated with 95% O_2_/5% CO_2_ containing (in mM): 92 NaCl, 20 HEPES, 25 glucose, 30 NaHCO_3_, 2.5 KCl, 1.2 NaPO_4_, 5 sodium ascorbate, 3 sodium pyruvate, and 2 thiourea (303–306 mOsm) at room temperature. Slices were allowed to incubate at room temperature in this solution for at least 1 h before being transferred to the recording chamber.

The recording chamber was kept at 32 °C and perfused with a pump (World Precision Instruments) at a flow rate of 1.5–2.0 ml/min with aCSF containing (in mM): 126 NaCl, 2.5 KCl, 1.4 NaH_2_PO_4_, 1.2 MgCl_2_, 2.4 CaCl_2_, 25 NaHCO_3_, and 11 glucose (303–305 mOsm) for recordings. Whole-cell recordings of intrinsic excitability were made utilizing glass microelectrodes (3–4 MΩ) containing (in mM): 135 K-gluconate, 10 HEPES, 4 KCl, 4 Mg-ATP, and 0.3 Na-GTP. Whole-cell recordings of evoked EPSCs were made utilizing glass microelectrodes (2–3 MΩ) containing (in mM): 117 cesium methanesulfonate, 20 HEPES, 0.4 EGTA, 2.8 NaCl, 5 TEA-Cl, 4 Mg-ATP, 0.4 Na-GTP, and 5 QX-314 (280–285 mOsm). To isolate EPSCs, cells were voltage clamped at −70 mV and 100 μM picrotoxin was included in the aCSF. For AMPAR current–voltage curves, 100 μM spermine was included in the internal solution and AP-5 (50 μM) and picrotoxin (100 μM) was included in the aCSF. MSNs were identified based on morphology, hyperpolarized membrane potential, and intrinsic characteristics (i.e., delayed spiking, rectification with hyperpolarizing current steps) [12]. Cells were identified using IR-DIC optics on an inverted Olympus BX5iWI microscope. TdTomato-positive cells were classified based on strong fluorescence, while TdTomato-negative cells lacked fluorescence but were adjacent to TdTomato-positive cells. Optogenetically-evoked synaptic responses were elicited using a 473 nm laser (1 ms pulse) directed at the brain slice (Thor Labs). Laser intensity was adjusted to evoke EPSCs at approximately half of maximal amplitude (1–10 mW). VH-evoked EPSCs were analyzed using ClampFit software (Molecular Devices). Neurons were voltage clamped at −70 mV utilizing a Multiclamp 700B amplifier (Molecular Devices). Data were filtered at 2 kHz and digitized at 20 kHz using a 1440A Digidata Digitizer (Molecular Devices). Series resistance (10–20 MΩ) was monitored using a −5 mV voltage step. Cells with >20% change in series resistance were discarded from further analysis.

### Quantification of VH ChR2-eYFP terminal fluorescence

Mice were injected with AAV1-CaMKII-ChR2-eYFP into the VH. Four weeks later mice underwent repeated footshock stress as described above. Twenty-four hours after day 4 fear recall test, mice were anaesthetized with Euthasol and subsequently transcardially perfused with saline and 4% paraformaldehyde. Brains were sectioned (50 μm thickness) on a Leica VT1200S vibratome, mounted on slides, and coverslipped utilizing Mowiol^®^ 4–88 containing DAPI. Confocal Z-stack images of the NAc were acquired utilizing an Olympus laser-scanning confocal microscope. All samples were imaged utilizing identical imaging parameters for comparison across treatment groups.

### Drugs

All drugs were purchased from Tocris, with the exception of picrotoxin, which was purchased from Abcam. Stock solutions of drugs for electrophysiology were dissolved in ddH_2_O and diluted to the final working concentration on the day of the experiment. Stock picrotoxin solution was prepared by gentle warming and kept at room temperature.

### Statistical analysis

All experiments were performed at least twice to avoid any unspecific day/condition effect and all comparisons relate test to control data from littermate animals collected during the same time period. After histological verification, mice with incorrect virus injection or fiber optic implantation were excluded from data analysis. Animals were randomized by cage before surgery and behavioral experiments. All experiments were conducted in a blinded manner such that assays were conducted and analyzed without knowledge of the specific manipulation being performed. All analyses were performed using the following softwares: Clampex, MiniAnalysis, Ethovision, Video freeze, Excel, and Matlab (Mathworks). All data are presented as mean values ± SEM performed by GraphPad Prism 6 software (Instat, GraphPad Software). Statistical significance was assessed by unpaired *t* test or two-way ANOVA. Alpha values were considered significant with a *p* < 0.05 and nonsignificant with a *p* > 0.05.

## Supplementary Material

Supplementary material
