# Supplementary material for: Cooperative synaptic and intrinsic plasticity in a disynaptic limbic circuit drive stress-induced anhedonia and passive coping in mice
Source: Mol Psychiatry. 2020 Mar 11;26(6):1860–79. doi: 10.1038/s41380-020-0686-8 (PMC7735389; doi:10.1038/s41380-020-0686-8)
Supplement: Supplementary file 1 — Supplemental methods [file 41380_2020_686_MOESM1_ESM.pdf]

Supplementary material

Bacterial and Virus Strains

AAV1-CaMKII-ChR2-eYFP	Penn Vector Core / Addgene	Cat #: 26969-AAV1
AAV1-EF1α-DIO-eYFP	Penn Vector Core	Available through Addgene
AAV9-EF1α-DIO-eYFP	Penn Vector Core	Available through Addgene
AAV1-Syn-GCaMP6s	Penn Vector Core	Available through Addgene
AAV9-Syn-FLEX-GCaMP6f	UNC Vector Core	Available through Addgene
AAV2-retro-CMV-IE-Nuc-iRFP-2A-iCre	NIDA Genetic Engineering and Viral Vector Core	N/A
AAV9-EF1a-DIO-Myc-Kir2.1 GYG144-146AAA E224G Y242F-P2A-dTomato-WPRE-bGHpA	Mingshan Xue	N/A
AAV1-EF1a-DIO-Myc-Kir2.1 E224G Y242F-T2A-tdTomato-WPRE-hGHpA	Mingshan Xue	N/A
AAV9-EF1a-DIO-Myc-Kir2.1 E224G Y242F-P2A-dTomato-WPRE-bGHpA	Mingshan Xue	N/A

Software and Algorithms

pClamp	Molecular Devices	N/A
Ethovision	Noldus	N/A
Image J	NIH	https://imagej.nih.gov/ij/download.html
Matlab	Mathworks	N/A
Prism	Graphpad	N/A
Matlab	Mathworks	https://github.com/djamesbarker/FiberPhotometry

Supplementary Figure Legends

Figure S1: As related to figure 1

A) Number of transitions between mobile and immobile states are not modified by footshock stress (n=7-8 per group; t-test; $t_{(13)}$=0.41, *p=0.69*).
B) Total distance traveled in the open field test does not differ between footshock and tone groups (n=6 per group; t-test; $t_{(10)}$=0.04, *p=0.97*).
C) Anxiety-like behavior in the elevated plus maze does not differ between footshock and tone groups (closed arm duration: n=6 per group; t-test; $t_{(10)}$=0.95, *p=0.37*); (Open arm frequency: n=6 per group; t-test; $t_{(10)}$=1.37, *p=0.2*); (velocity: n=6 per group; t-test; $t_{(10)}$=0.89, *p=0.4*).
D) No group differences in transitions (n=6 per group; t-test; $t_{(10)}$=1.15, *p=0.28*) or percentage of time spent in the light side (n=6 per group; t-test; $t_{(10)}$=0.28, *p=0.79*) in the light-dark box test.

Figure S2: As related to figure 2

E) Locations of fiber tips in tone control (black) and footshock stress (red) groups in NAcmSh for the photometry recordings of VH to NAcmSh.

Figure S3: As related to figure 3

A) Footshock stress does not modify ChR2-eYFP expression in the NAc (n=5 per group; t-test; $t_{(8)}$=0.64, *p=0.54).*

Figure S4: As related to figure 3

A) Experimental timeline.
B) In-vivo long-term depression of VH afferents to the NAc does not modify freezing to the context or cue on the test day (n=10 per group; Two-way ANOVA; Virus Main Effect; $F_{(1, 19)}$=0.09012, *p=0.7673;* Time Main Effect; $F_{(14, 266)}$=9.366, *p<0.0001;* Treatment x Time Interaction; $F_{(14, 266)}$=0.3758, *p=0.9807).*
C) Locations of fiber tips in tone control (black) and footshock stress (red) groups in NAcmSh for the *in vivo* LTD experiment of VH to NAcmSh.

Figure S5: As related to figure 5

A) Input-output curves and rheobase of action potential firing in D1-MSNs from control (white) and footshock stress mice (blue) in the presence of excitatory (DNQX; AP-5) and inhibitory (picrotoxin) ionotropic receptor antagonists. Input-output curves (n=13-17 per group; Two-way ANOVA; Treatment x Current Input Interaction; $F_{(10,280)}$=7.76, *p<0.0001).*
B) Rheobase in footshock stress and control mice (n=13-17; t-test; $t_{(28)}$=4.78, *p<0.0001).*
C) Resting membrane potential is not affected in D1 MSNs from mice exposed to repeated footshock stress relative to controls (D1 MSNs; n=8-11; t-test; $t_{(17)}$=0.71, *p=0.49*).

Figure S6: As related to figure 6

A) Locations of fiber tips in tone control (black) and footshock stress (red) groups in NAcmSh for the photometry recordings of Dyn-expressing neurons located within the NAcmSh.

Figure S7: As related to figure 7

A) Schematic of virus injection in ProDyn icre mice (left) and representative traces of voltage responses to current injection and evoked action potentials in cells infected with either AAV_1-EF1α-DIO-eYFP or AAV_1-EF1α-DIO-$KIR_{2.1}$-2A-tdtomato (right).

B) Input resistance from infected D1 MSNs from ProDyn iCre mice infected with AAV_1-EF1α-DIO-eYFP or AAV_1-EF1α-DIO-$KIR_{2.1}$-2A-tdtomato expression. (n=6-6; t-test; $t_{(10)}=7.34$, *p<0.0001*).

C) IRK currents from infected D1 MSNs from ProDyn iCre mice infected with AAV_1-EF1α-DIO-eYFP or AAV_1-EF1α-DIO-$KIR_{2.1}$-2A-tdtomato expression (t-test of peak current; n=6 per group; t-test; $t_{(10)}=3.61$, *p=0.0048).*

D) Schematic of virus injection in ProDyn iCre mice (left) and representative traces of voltage responses to current injection and evoked action potentials in cells infected with either AAV_9-EF1α-DIO-eYFP or AAV_9-EF1α-DIO-$KIR_{2.1}$-2A-tdtomato (right).

E) Input resistance from infected D1 MSNs from ProDyn iCre mice infected with AAV_9-EF1α-DIO-eYFP or AAV_9-EF1α-DIO-$KIR_{2.1}$-2A-tdtomato expression. Expression of DN-KIR2.1 in D1 MSNs decreases input resistance (n=9-10; t-test; $t_{(17)}=3.73$, *p=0.0017*).

F) Expression of DN-KIR2.1 in D1 MSNs decreases IRK currents (t-test of peak current; n=6-14 per group; t-test; $t_{(18)}=5.86$, *p<0.0001)*.

Figure S8: As related to figure 7

A) Distance traveled (n=4-7; Two-way ANOVA; Treatment Main Effect; $F_{(1, 9)}=0.77$, *p=0.4;* Time Main Effect; $F_{(2, 18)}=28.42$, *p<0.0001;* Treatment x Time Interaction; $F_{(2, 18)}=1.49$, *p=0.25*) and duration of time spent in the center (Two-way ANOVA; Treatment Main Effect; $F_{(1, 9)}=2.09$, *p=0.18;* Time Main Effect; $F_{(2, 18)}=3.32$, *p=0.06;* Treatment x Time Interaction; $F_{(2, 18)}=0.1$, *p=0.91*) of an open field maze in a separate cohort of ProDyn iCre mice expressing Cre-dependent eYFP or KIR2.1.

B) Closed arm duration in the elevated plus maze in ProDyn iCre mice expressing Cre-dependent eYFP or KIR2.1 (n=4-7; t-test; $t_{(9)}=1.73$, *p=0.12*)

C) Latency to enter the dark compartment in the light/dark box test (n=4-7; t-test; $t_{(9)}=0.62$, *p=0.55*)

Figure S1

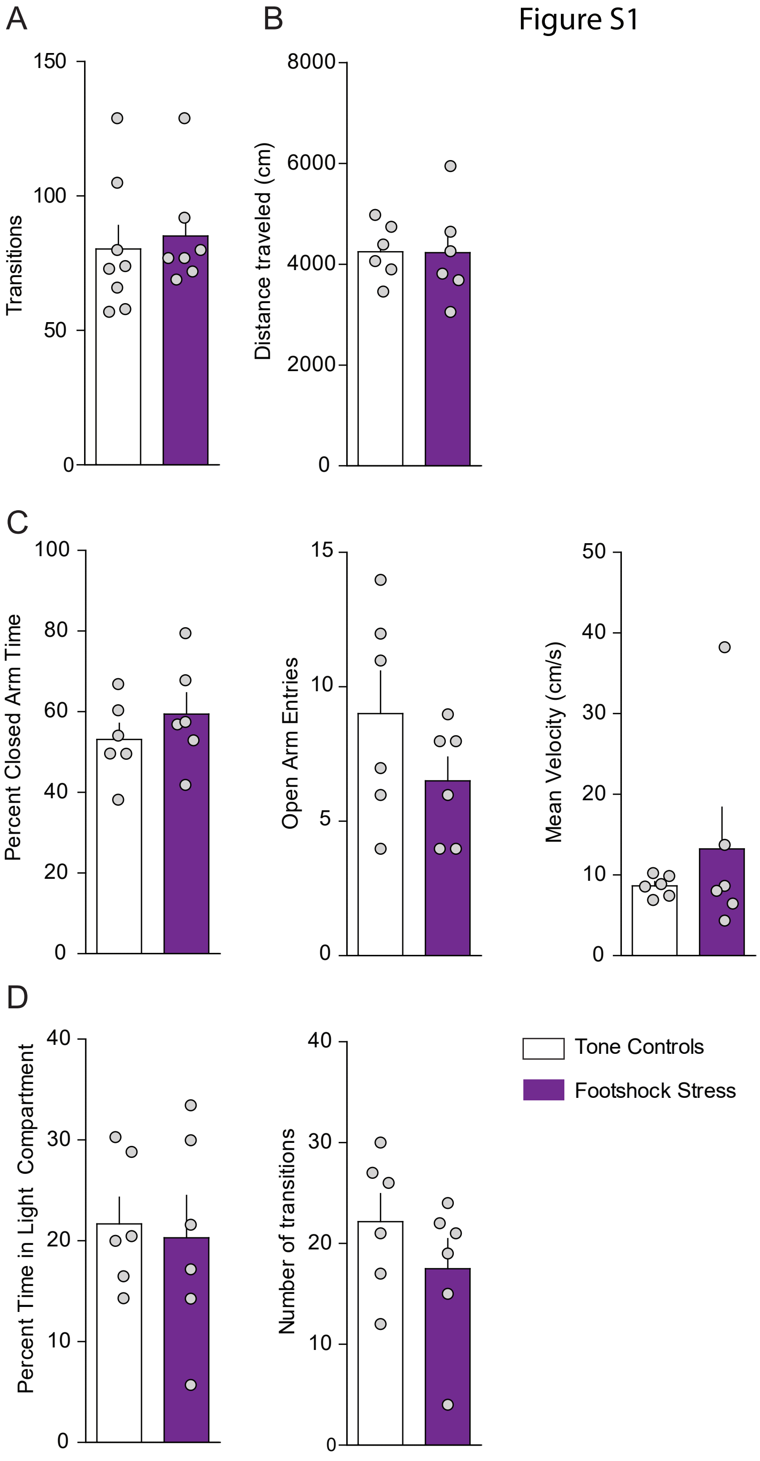

A
Transitions
150
100
50
0
B
Distance traveled (cm)
8000
6000
4000
2000
0
C
Percent Closed Arm Time
100
80
60
40
20
0
Open Arm Entries
15
10
5
0
Mean Velocity (cm/s)
50
40
30
20
10
0
D
Percent Time in Light Compartment
40
30
20
10
0
Number of transitions
40
30
20
10
0
Tone Controls
Footshock Stress

Figure S2

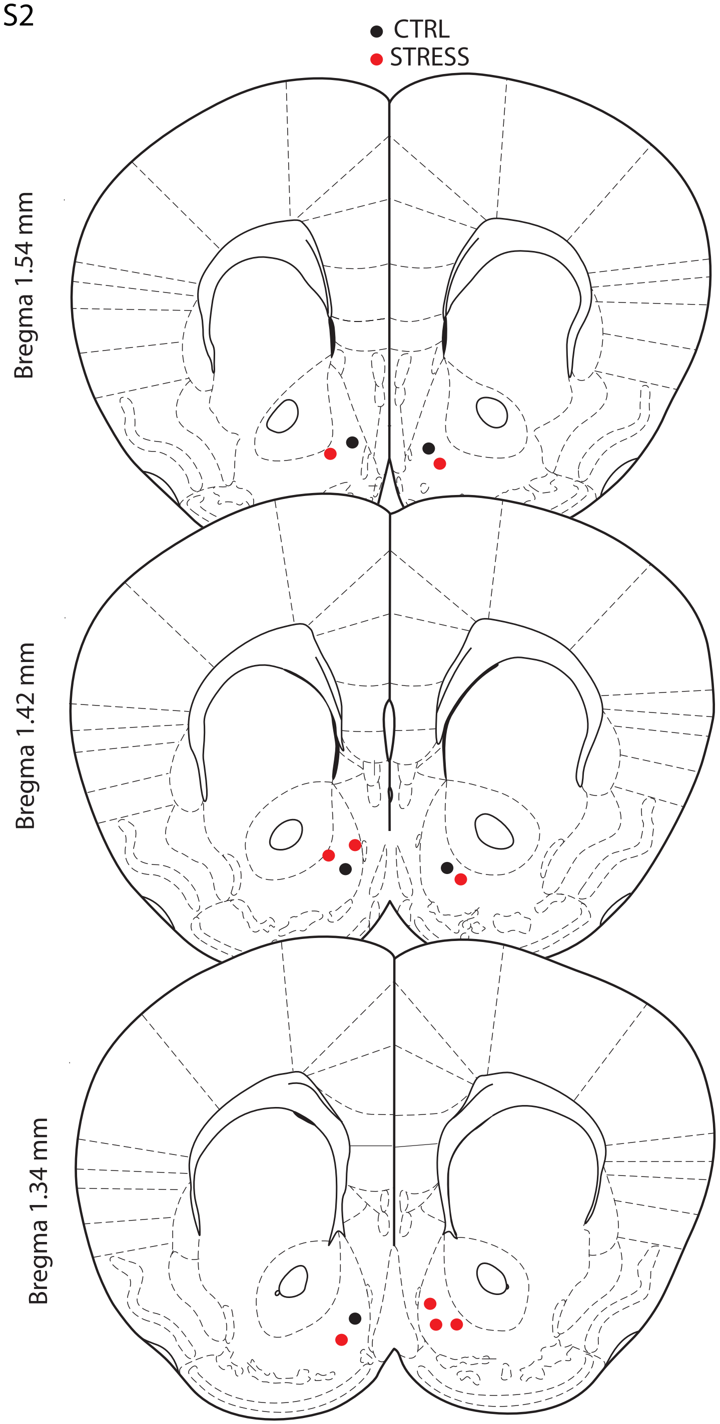

Figure S3

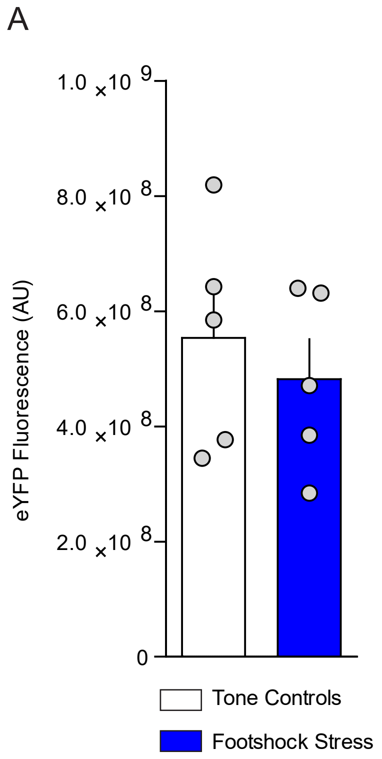
A
eYFP Fluorescence (AU)
1.0 ×10 9
8.0 ×10 8
6.0 ×10 8
4.0 ×10 8
2.0 ×10 8
0
Tone Controls
Footshock Stress

Figure S4

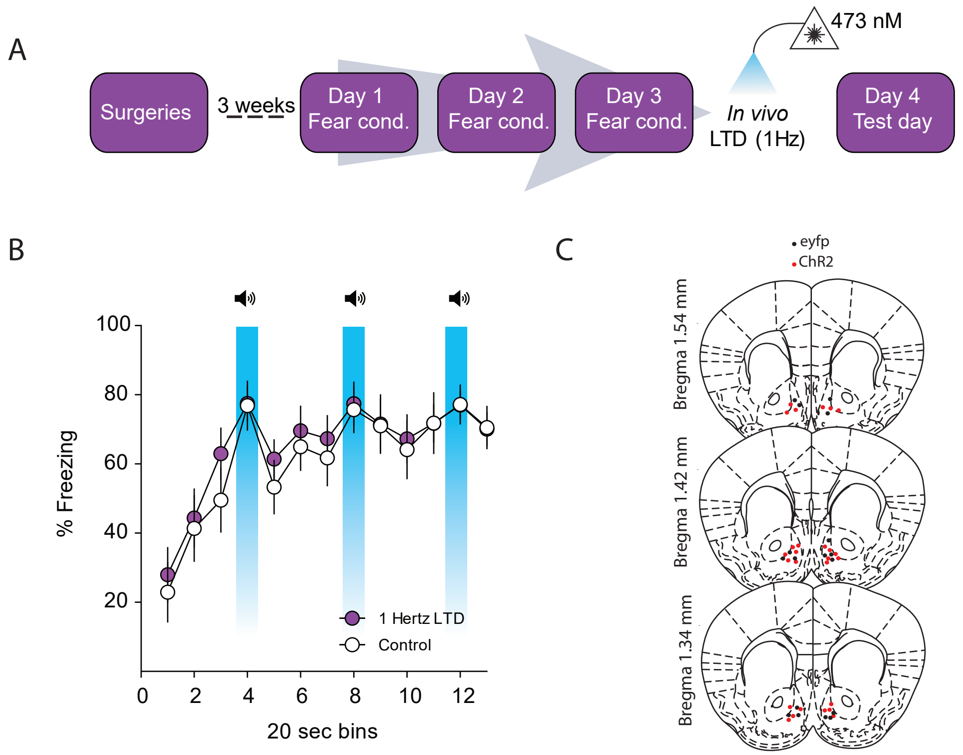

A
Surgeries
3 weeks
Day 1
Fear cond.
Day 2
Fear cond.
Day 3
Fear cond.
473 nM
In vivo
LTD (1Hz)
Day 4
Test day
B
100
80
60
40
20
% Freezing
0
2
4
6
8
10
12
20 sec bins
1 Hertz LTD
Control
C
eyfp
ChR2
Bregma 1.54 mm
Bregma 1.42 mm
Bregma 1.34 mm

Figure S5

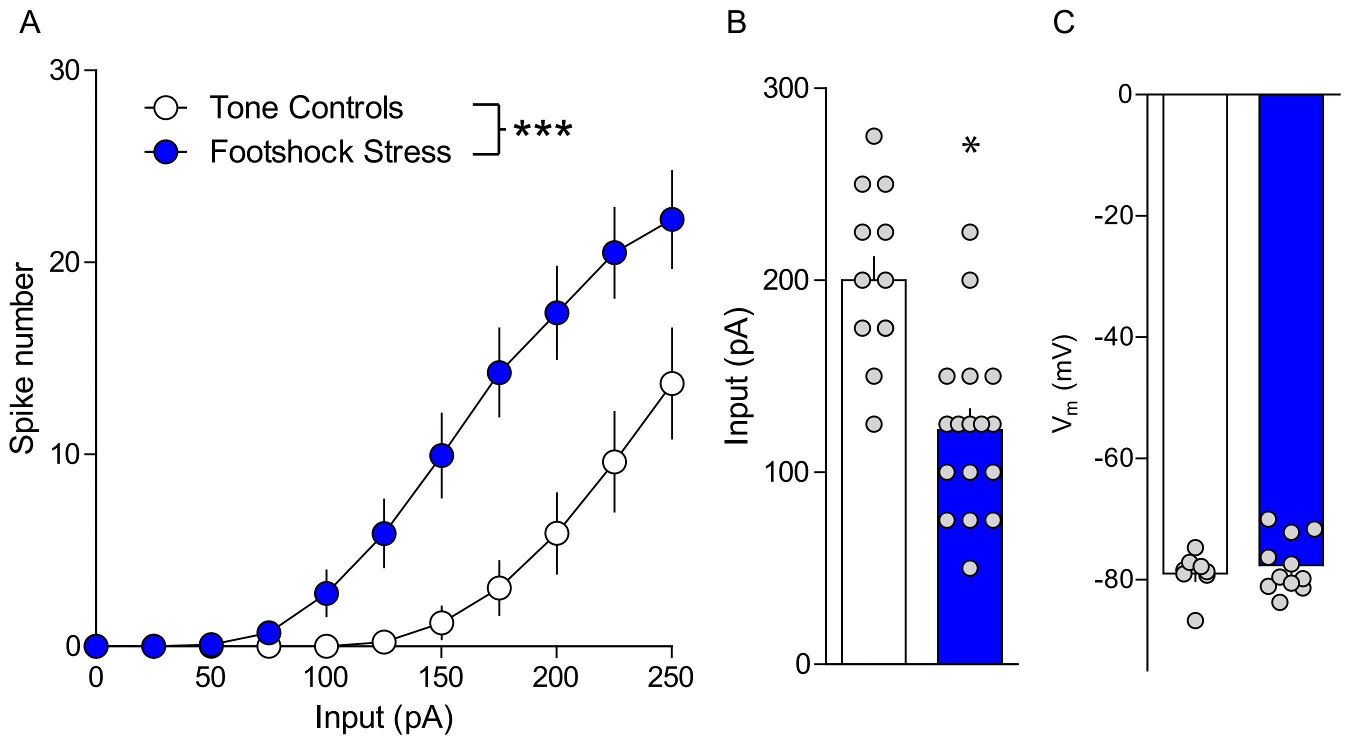
A
B
C
Tone Controls
Footshock Stress
***
Spike number
Input (pA)
0
50
100
150
200
250
0
10
20
30
Input (pA)
300
200
100
0
*
V_m (mV)
0
-20
-40
-60
-80

Figure S6

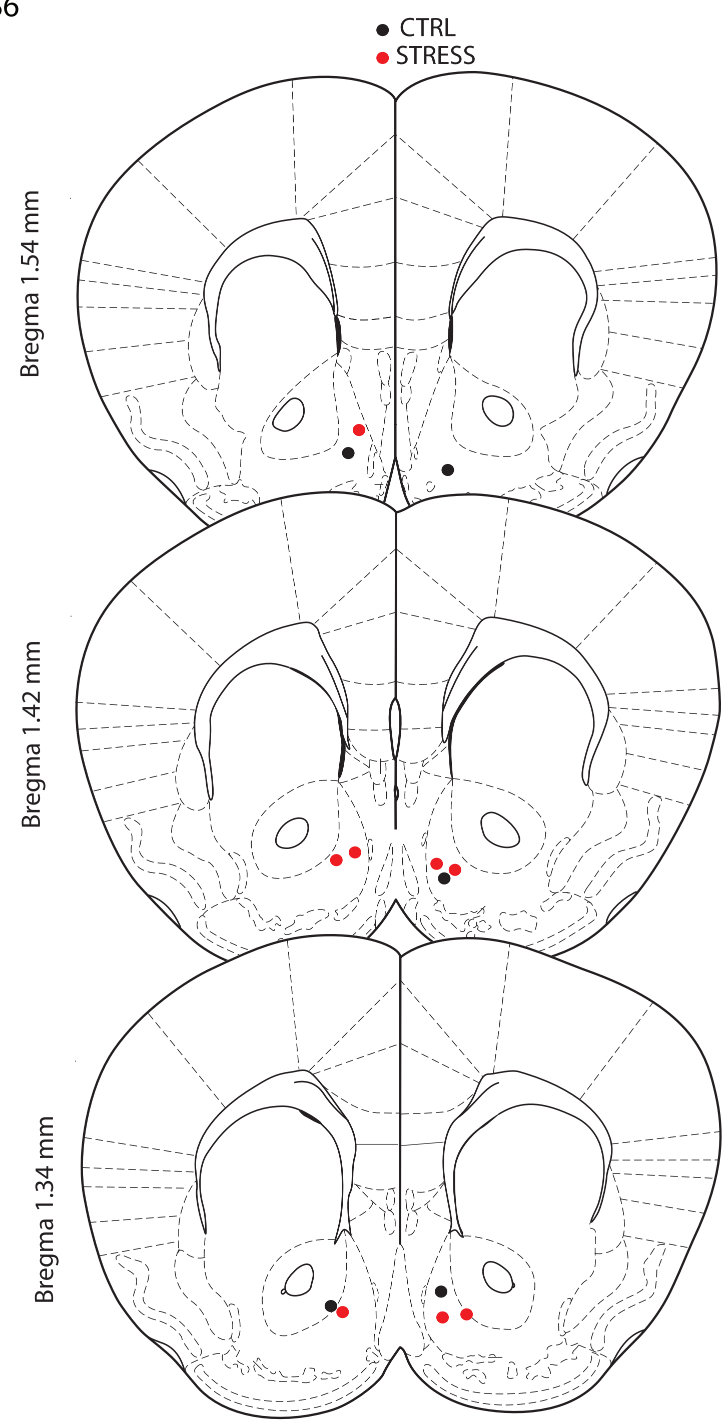

Figure S7

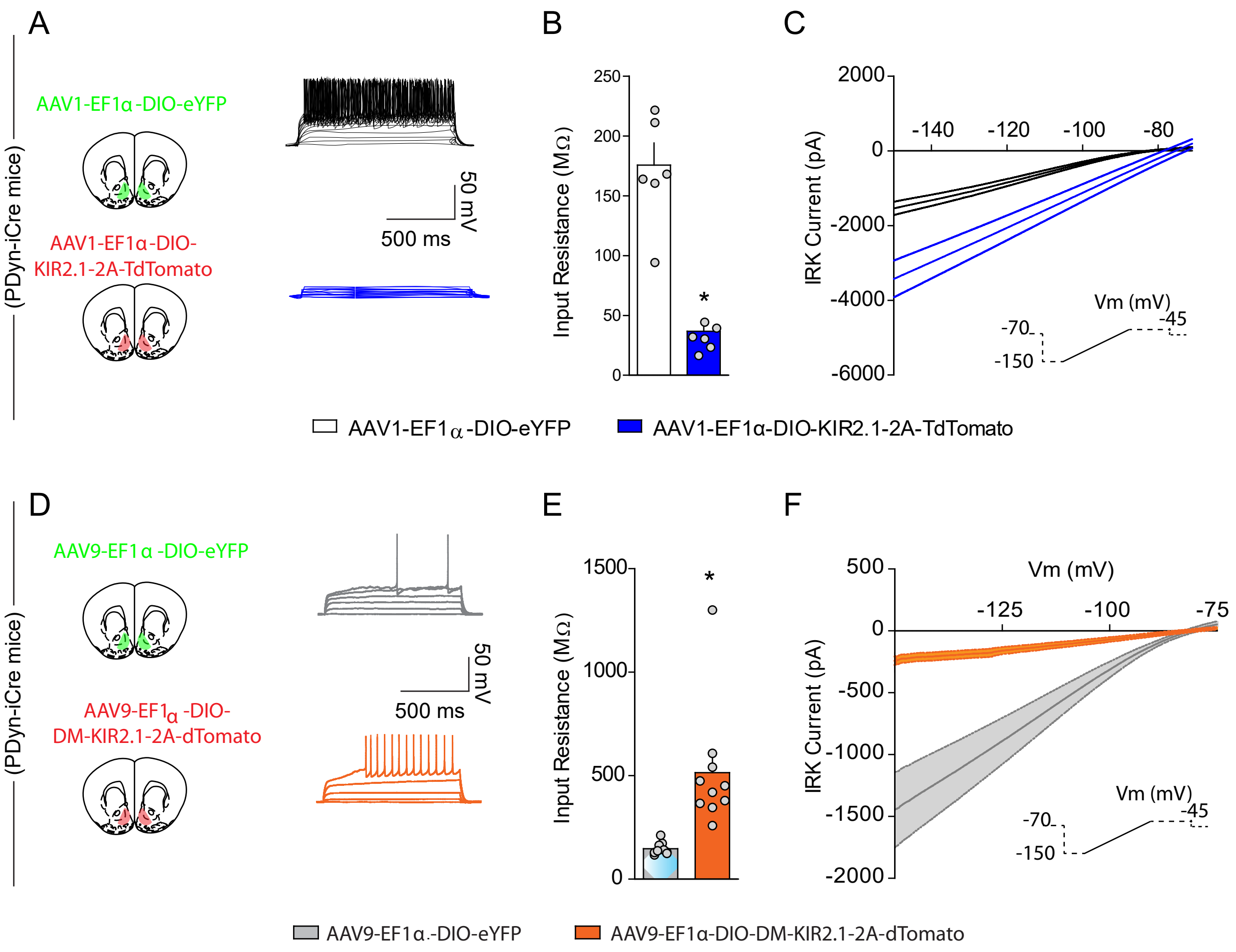

A
(PDyn-iCre mice)
AAV1-EF1α-DIO-eYFP
AAV1-EF1α-DIO-KIR2.1-2A-TdTomato
50 mV
500 ms
B
Input Resistance (MΩ)
250
200
150
100
50
0
*
C
IRK Current (pA)
2000
0
-2000
-4000
-6000
-140
-120
-100
-80
Vm (mV)
-70
-150
-45
AAV1-EF1α-DIO-eYFP
AAV1-EF1α-DIO-KIR2.1-2A-TdTomato
D
(PDyn-iCre mice)
AAV9-EF1α -DIO-eYFP
AAV9-EF1α -DIO-DM-KIR2.1-2A-dTomato
50 mV
500 ms
E
Input Resistance (MΩ)
1500
1000
500
0
*
F
IRK Current (pA)
Vm (mV)
500
0
-500
-1000
-1500
-2000
-125
-100
-75
Vm (mV)
-70
-150
-45
AAV9-EF1α.-DIO-eYFP
AAV9-EF1α-DIO-DM-KIR2.1-2A-dTomato

Figure S8

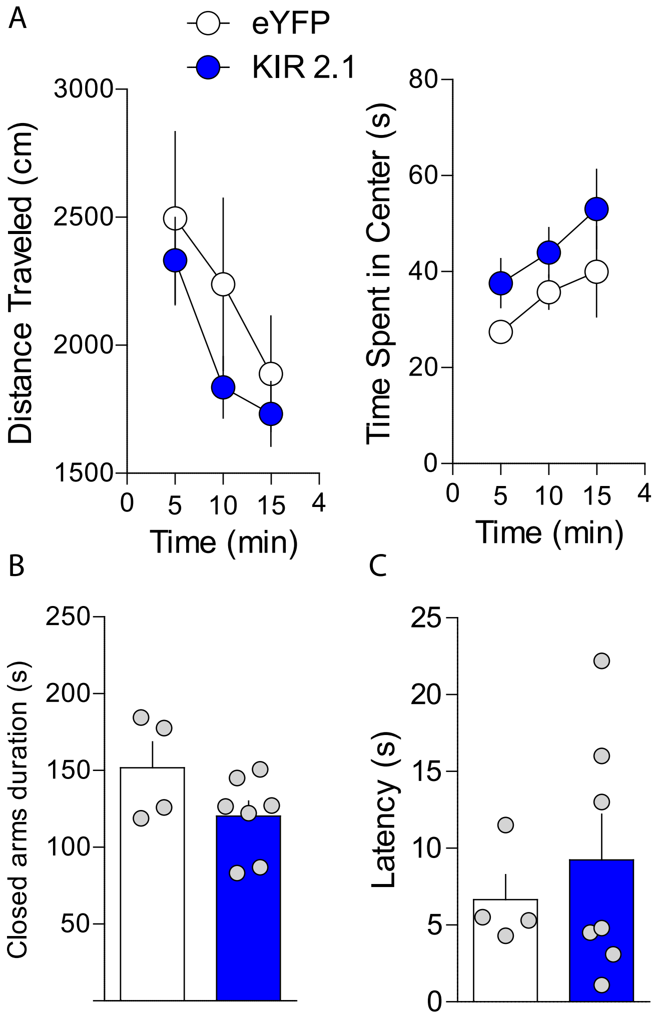

A
eYFP
KIR 2.1
3000
2500
2000
1500
Distance Traveled (cm)
0
5
10
15
4
Time (min)
80
60
40
20
0
Time Spent in Center (s)
0
5
10
15
4
Time (min)
B
250
200
150
100
50
Closed arms duration (s)
C
25
20
15
10
5
0
Latency (s)
